# Tailoring AI and ML models for genotype-by-environment prediction leveraging environmental covariates: A European rye example

**DOI:** 10.1007/s00122-026-05280-z

**Published:** 2026-07-15

**Authors:** Wera Eckhoff, Florence Parat, Gennady Bracho-Mujica, Clemens Flamm, Daniela Bustos-Korts, Hans-Peter Piepho

**Affiliations:** 1 Biostatistics Unit, Institute of Crop Science, University of Hohenheim, Stuttgart, Germany; 2 KWS SAAT SE & Co. KGaA, Einbeck, Germany; 3 Österreichische Agentur Für Gesundheit Und Ernährungssicherheit GmbH, Vienna, Austria; 4 Faculty of Agricultural and Food Sciences, Universidad Austral de Chile, Valdivia, Chile

## Abstract

***Key messages*:**

We explored target variable and loss function engineering for isolating genotype-by-environment interactions from main environmental effects to improve machine learning and deep neural network-based prediction of genotype performances using environmental covariates to make inference for untested locations and years.

**Abstract:**

Accurate prediction of environment-specific genotype performance remains a major challenge in crop improvement and agricultural decision-making. Machine-learning (ML)- and deep neural network (DNN)-based predictions are often dominated by environmental main effects rather than genotype-by-environment interaction (GxE) effects, which hinders their adoption in plant breeding. This study aims to improve GxE predictive modeling in rye but also generally across crops by developing novel approaches to tailor ML and DNN models toward predicting environment-specific genotype differences and rankings rather than absolute performances. We introduce two methodologies: (1) target-variable engineering based on linear mixed-model decompositions to isolate GxE effects and (2) a custom-loss-function implementation of the mean squared error of differences (Piepho 1998) to optimize models directly for prediction of within-environment genotype differences. The motivating dataset covers major rye growing regions worldwide and models were evaluated using a comprehensive cross-validation (CV) framework. Our approaches improved predictive abilities of ML/DNN models by + 21 to + 62% compared to classical ML/DNN-based yield prediction. We increased predictive accuracy for environment-specific genotype rankings between + 15.0% and + 9.8% across different CV schemes over baseline genotypic main effects. Furthermore, historical weather records enabled prediction of genotype performances in future, untested years. This work demonstrates that tailored ML and DNN strategies can outperform classical methods for GxE prediction, offering practical value for plant breeders, growers and variety testing authorities.

**Supplementary Information:**

The online version contains supplementary material available at 10.1007/s00122-026-05280-z.

## Introduction

Genotype-by-environment interaction (GxE) causes substantial variation in rankings of genotypes within environments even in relatively stable crops like hybrid rye (Wilde and Miedaner 2021). Climate change is projected to shift approximately 5 to 13% of global land surface into a different climate classification by the end of the century (Beck et al. 2023) and to increase spatial heterogeneity of crop productivity within the target population of environments (TPE; Challinor et al. 2014; Kersebaum and Nendel 2014). This trend poses a significant challenge to conventional broad-adaptation selection strategies, particularly in hybrid rye (Riedesel et al. 2024), where material from the same breeding pipeline is often supplied to markets ranging from Canada to Russia. Tools for accurate prediction of GxE can enable breeders to exploit specific genotype adaptations in a targeted manner, thereby contributing to secure crop yields under the rising influence of climate change.

Next to various mixed-model-based approaches, like environmental-kinship-based genomic best linear unbiased prediction (E-GBLUP; Jarquín et al. 2014), factorial regression (Denis 1988; Denis et al. 1997) or factor-analytic models (Tolhurst et al. 2022; Piepho and Blancon 2023), also artificial intelligence (Jubair et al. 2023; Kick et al. 2023) and machine-learning (ML) models (Westhues et al. 2021; Fernandes et al. 2024; He et al. 2025) are explored increasingly for the purpose of GxE prediction based on environmental data. However, deep neural networks (DNN) and other ML models are often driven by environmental main effects rather than by genotypic main and GxE interaction effects. This occurs because most multi-environment trial (MET) datasets exhibit a substantially larger proportion of the total variance explained by environmental factors than by genotype-related factors (Laidig et al. 2017; Kick and Washburn 2023; Avagyan et al. 2025). Consequently, ML and DNN models optimized using mean square error (MSE) or mean absolute error (MAE) for predicting environment-specific genotype yields tend to focus on accurately predicting average yields per environment, while providing comparatively poor resolution of genotype rankings within environments (Washburn et al. 2024).

Firstly, we hypothesized that variance dissection via linear mixed models can be leveraged to engineer GxE target variables by removing any environment main effects from the response-variable grain dry matter yield (GDY). Other researchers in the field of predictive breeding have explored various response-variable-engineering approaches. For instance, Montesinos-Lopez et al. (2023) reformulated genomic selection from a regression problem into a binary classification task. A related concept—though without explicit target-variable engineering—can also be found in Heslot et al. (2013), who proposed two separate prediction models, one for the genetic main effect and another for the GxE deviation, which were then combined to build the within-environment phenotype predictions. Likewise, Avagyan et al. (2025) picked up the idea to factor out the main environmental effects by modeling them as fixed in a penalized factorial regression, enabling the estimation of genotype sensitivities in response to environmental covariates only for the GxE component. Although response-variable engineering has been examined in previous studies, to the best of our knowledge no prior work has described the use of linear mixed model (LMM)-derived linear combinations of estimated GxE effect coefficients as target variables in ML or DNN models.

Secondly, we hypothesized that custom loss functions based on the mean squared error of pairwise genotype differences (MSED) within environments can disentangle environment and genotype-related components directly within the ML- and DNN-model optimization process. This loss function allows models to learn from residuals of genotype differences rather than from residuals of genotype absolute yields. Custom loss functions in ML and DNN models are increasingly used in other research domains (Barton et al. 2021; Wu et al. 2023; Xiong et al. 2023; Drusinsky et al. 2024). A similar loss function to the MSED was previously formulated by Drusinsky et al. (2024) in the context of RNA expression level prediction from DNA sequence data in humans. In the field of plant breeding loss function engineering remains rare. Prior studies have applied the MSED as a post-prediction evaluation metric (Piepho 1998; Studnicki et al. 2017; Buntaran et al. 2021; Tadese et al. 2024), but have not integrated it directly into model optimization. To our knowledge, this is the first study to evaluate and demonstrate the usefulness of MSED as a custom loss function for GxE prediction.

Thirdly, another major challenge in GxE prediction is that for many relevant use cases, the actual set of weather variables is not available at the time of decision-making. Furthermore, predictability of the next season’s weather conditions (i.e., seasonal forecast) is limited. For example, growers face this issue whenever making variety-purchase decisions. Following de los Campos et al. (2020) and Gillberg et al. (2019), we adopted the approach of using recent historical weather data as a proxy for future conditions and as an indicator of the climatic characteristics at a given site (Table 1).

**Table 1:** Overview of the most important abbreviations

Abbreviation	Definition
GxE	Genotype-by-environment interaction
GDY	Grain dry matter yield
MAE	Mean absolute error
MSE	Mean squared error
MSED	Mean squared error of differences
PCC	Pearson correlation coefficient
SCC	Spearman correlation coefficient
LMM	Linear mixed model
REML	Residual maximum likelihood
E-BLUP	Environmental-kinship-based genomic best linear unbiased prediction
ML	Machine learning
GBA	Gradient boosting algorithms
XGB	XGBoost, Python package and gradient boosting algorithm
LGBM	LightGBM, Python package and gradient boosting algorithm
DNN	Deep neural network
yHatGE & yHatGGE	Designed GxE target variables proposed in this study
envCV	Unique combination of year and location
E	Leave-one-envCV-out cross-validation
L	Leave-one-location-out cross-validation
Y	Leave-one-year-out cross-validation
LY	Leave-one-year-and-one-location-out cross-validation
TPE	Target population of environments
MET	Multi-environment trial
OVT	Official variety trials
PRT	Post-release trials
VP	VorsprungPlus, KWS internal pre-commercial test network

To demonstrate our developed methodology, we assembled one of the most comprehensive hybrid rye grain yield datasets to date, encompassing trials across Germany, Poland, Denmark, Austria and Czech Republic—and a test set consisting of trials from the two biggest producer countries (Germany and Poland) accounting for about 43% of global rye production (Helgi-Library 2023; USDA 2024) Therefore, this study also addresses the scarcity of GxE research in rye with few notable examples (Laidig et al. 2017; Hadasch et al. 2020).

## Materials and methods

### Data description

#### Phenotypic data

We assembled an extensive rye grain yield dataset across the core growing areas in Central Europe with ~ 23 k data points of trial-wise best linear unbiased estimates (BLUEs) of genotype means for GDY from eight different trial networks. No pedigree or molecular marker information was available in this study. Thus, the presented methodology focuses on exploiting environmental covariates and phenotypic patterns when modeling G × E interactions. The dataset comprises trials from 2013 to 2024 in five European countries accounting for approximately 60% of world rye production (USDA 2024). Our training data include official variety testing trials (OVT) from all five countries conducted by the Austrian Agency for Health and Food Safety (AGES) in Austria, the Central Institute for Supervising and Testing in Agriculture (ÚKZÚZ) in Czech Republic, the German Federal Office of Plant Varieties (BSA) in Germany, TystofteFonden in Denmark and the Research Center for Cultivar Testing (COBORU) in Poland. Details of the country-wise dataset dimensions can be found in Table 2. We also integrated data from a pre-commercial trial network of KWS SAAT SE & Co. KGaA (VP), conducted in Germany and Poland since 2016. For OVTs from Austria, Germany and Poland as well as the VP trials, we only used results from KWS genotypes due to data confidentiality reasons.

**Table 2:** Dimensions of the phenotypic input dataset and its different trial series across countries, namely Austria (AT), Czech Republic (CZ), Germany (DE), Denmark (DK) and Poland (PL)

Country	Trial^a^	Observations	Treatments	Genotypes	Environments	Locations	Years
AT	OVT/PRT	649	2	50	37	11	2018–2021, 2023, 2024
CZ	OVT	646	2	25	21	9	2020, 2021, 2023
DE	OVT	5590	2	97	208	33	2017–2024
DK	OVT	670	1	152	21	9	2017–2021, 2023, 2024
PL	OVT	2680	2	82	60	11	2018–2020, 2022–2024
DE	PRT	4787	2	44	462	72	2013–2023
DK	PRT	600	1	49	33	17	2017–2021
DE	VP^b^	6465	2	96	137	33	2016–2024
PL	VP^b^	904	2	98	18	4	2016–2024
Total		22991	2	223	858	167	2013–2024

^a^ OVT = Official variety trials of the respective national variety testing authority; PRT = post-release trials that correspond to so called Landessortenversuche (LSV) in AT and DE and National trials in DK; VP = VorsprungPlus, KWS internal pre-commercial test network

^b^ All trial networks are used in the training set and validation set. The VP trial network is used as test set as well

Our training set also includes post-release trial (PRT) data from Austria, Germany and Denmark. In Austria, PRT trials correspond to the Landessortenversuche (LSV), which are authorized by AGES and performed in coordination with the OVT. Furthermore, the federal state Lower Austria (Land-NÖ) conducts PRT trials that are managed by agricultural schools. In Germany, the LSV trials are conducted by the German Federal Agricultural Chambers. A detailed list of all contributing Chambers can be found in Table S1. LSV trials aim to provide a regionally adapted variety recommendation to farmers. In Denmark, PRT corresponds to “Landsforsøgene” conducted by SEGES Innovation and TystofteFonden, which follow a similar concept as LSV trials in Germany.

**Fig. 1:**
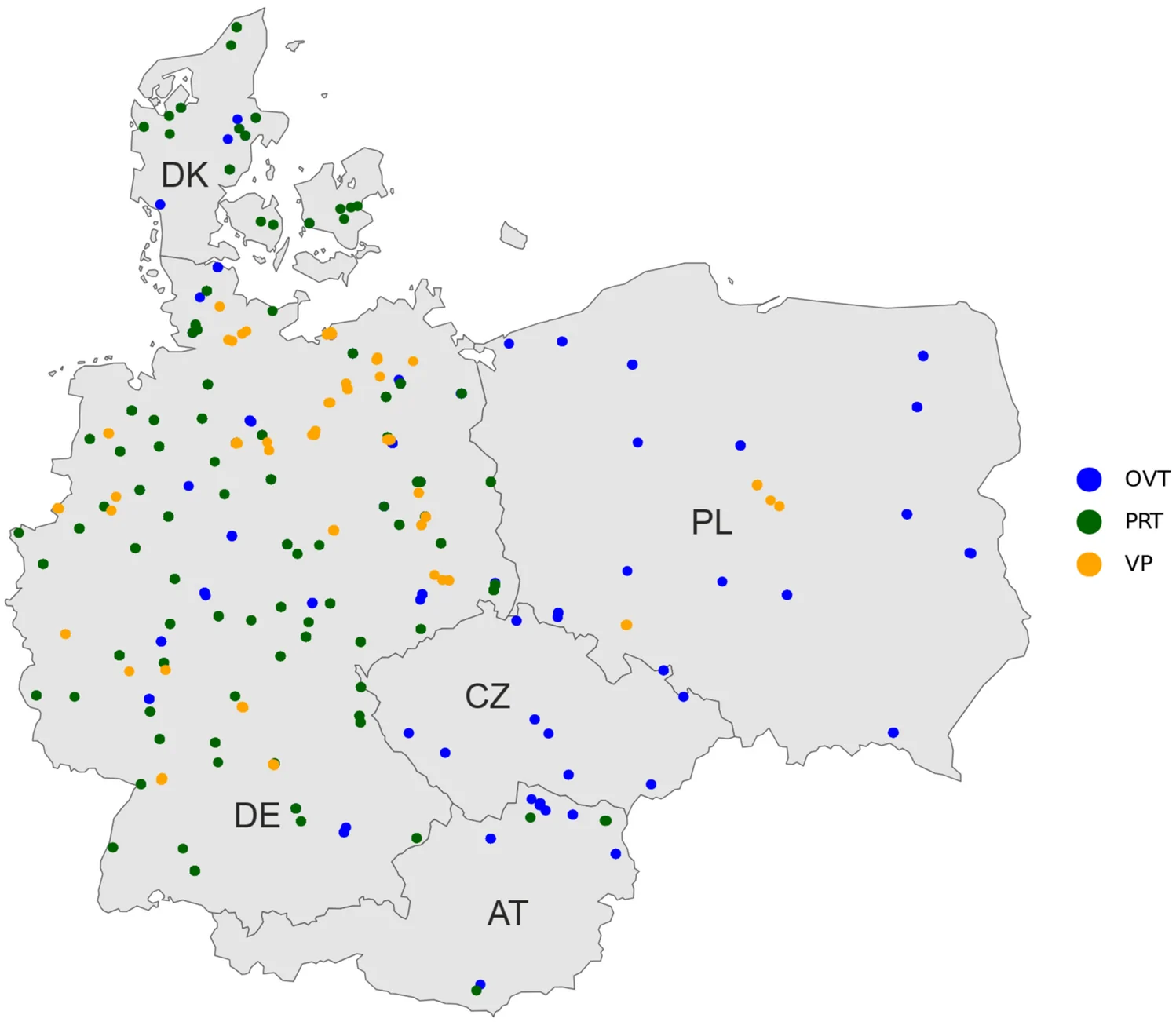
Overview of spatial distribution of trials across countries Austria (AT), Czech Republic (CZ), Germany (DE), Denmark (DK) and Poland (PL). The different trial networks nested within countries are official variety trials (OVT) in blue, post-release trials (PRT) in green and a KWS pre-commercial trial network (VP) in orange

Results from two different management regimens were available as indicated in Table 2. They will be referred to as intensive and extensive treatment hereafter. While under the intensive treatment plant protection agents and plant growth regulator were applied, this was not the case under the extensive treatment. At both treatment levels synthetic fertilizers were applied. The spatial distribution of trials across the five countries is shown in Fig. 1. An extended overview of the data sources, their authors and their availability can be found in Table S1. For the aforementioned trial networks, we obtained data as single-trial genotype BLUEs and not on plot level basis. Additionally, we retrieved the first year of OVT testing across the five countries in the training set from CPVO Variety Finder database (Community Plant Variety Office; CPVO) as a proxy for the age of a genotype.

#### Definition of terms environment and year

In this study we refer to an environment as the unique combination of factors year, location, trial and management regime (treatment). Genotypes from the same environment were grown under the same weather and soil conditions, as well as agricultural management regime. The factor “trial” was included in the definition of the factor “environment” because there are several trials per location and year in some cases. Due to the structure of the data, we defined an additional term, *envCV*, which represents the unique combination of year and location and is the unit used to define the cross-validation (CV) splits. Furthermore, we considered the year as the harvest year of the cultivation season, spanning from 1st September to 31st of August of the following calendar year.

#### Environmental data

Interpolated soil data were obtained on a coordinate basis from the open-source provider ISRIC Soilgrids database (Poggio et al. 2021). Soilgrids data points are available at 250 m spatial resolution. The soil data comprise 12 different parameters in five layers of depth between 0 and 100 cm. Interpolated daily weather data were obtained from DTN (DTN-ClearAg^TM^) for all trial coordinates in training set with 15 km spatial resolution for all years in the training set (2013 to 2024). Tables S3 and S4 give an overview of all weather and soil parameters that we used in this study. For VP trials, we had access to true field coordinates for most trials. For most OVT and PRT trials, only the location name was known, and therefore, the coordinates were randomly assigned to arable land at the respective location using the CORINE Land Cover data from 2018 at a 100-m raster-layer resolution (European Environment Agency 2020). We also used the CORINE Land Cover to check for land-use and identify coordinates located in, e.g., settlements, forest, pasture or water bodies and move them to the nearest arable-land coordinates. It needs to be taken into account that this approach limits the quality of environmental data that we obtained on a coordinate basis as will be further discussed in Sect.  4.3. DTN interpolated daily weather data were subject to DTN in-house quality control (DTN-ClearAg™). Additionally, five daily cumulated precipitation values over all twelve years were cut off at > 350 mm, since values in excess of this were considered unrealistic.

### Data preparation

#### Variance component analysis

Data points from all trial networks were available as design-effect adjusted single-trial genotype BLUEs. Stage-one analysis was performed by the respective trial-conducting organizations (Table S1).We then fitted the following LMM on stage-one BLUEs using ASReml-R 4.2 (Butler DG 2023) to estimate the variance components in the phenotypic data:

1 $$\begin{aligned} M_{inhjpk}^{\left( 1 \right)} = & \mu + t_{k} + c_{n} + z_{h} + l_{j} + \left( {zl} \right)_{hj} + \left( {zlv} \right)_{hjp} + \left( {zt} \right)_{hk} + \left( {lt} \right)_{jk} \\ & + \left( {zlt} \right)_{hjk} + \left( {zlvt} \right)_{hjpk} + g_{i} + \left( {gt} \right)_{ik} + \left( {gz} \right)_{ih} + \left( {gl} \right)_{ij} \\ & + \left( {gzl} \right)_{ihj} + \left( {gzlv} \right)_{ihjp} + \left( {gzlt} \right)_{ihjk} + e_{inhjpk} \\ \end{aligned}$$

where *μ* represents the fixed intercept, $t_{k}$ is the fixed treatment main effect, $c_{n}$ is the fixed country main effect, $z_{h}$ is the random year main effect, $l_{j}$ is the random location main effect, $\left( {zl} \right)_{hj}$ is the random interaction effect of year and location, $\left( {zlv} \right)_{hjp}$ is the random trial effect nested within year and location, $\left( {zt} \right)_{hk}$*,* $\left( {lt} \right)_{jk}$, $\left( {zlt} \right)_{hjk}$ and $\left( {zlvt} \right)_{hjpk}$ are the respective random interaction effects between treatment $t_{k}$ and year, location, the year–location interaction and the nested trial effect, $g_{i}$ is the random genotype main effect, $\left( {gt} \right)_{ik}$, $\left( {gz} \right)_{ih}$, $\left( {gl} \right)_{ij}$, $\left( {gzl} \right)_{ihj}$ and $\left( {gzlv} \right)_{ihjp}$ are the random interactions of genotype with treatment, year, location, the year–location interaction and the nested trial effect, $\left( {gzlt} \right)_{ihjk}$ is the random interaction effect between genotype, year, location and treatment and $e_{{{\mathrm{inhjpk}}}}$ is the random residual error associated with the stage-one BLUEs $M_{inhjpk}^{\left( 1 \right)}$ [$e_{inhjpk} \sim N(0,\sigma_{e}^{2})$]. First-stage variance–covariance matrices or first-stage standard errors of genotype BLUEs were not available. Hence, a constant residual variance $\sigma_{e}^{2}$ was assumed and weighting of stage-one BLUEs was not possible in our second-stage model (1). Therefore, in the second-stage analysis the highest order interaction term $\left( {gzlvt} \right)_{ihjpk}$ is confounded with the residual error term and was not included in model (1). We defined the variance explained by the interaction between the year–location nested trial and genotype $\left( {gzlv} \right)_{ihjp}$ as well as the residual error variance as unexplained GxE variances. The reason is that most coordinates could only be obtained on a location basis, not on a field trial basis. Therefore, the resolution of the environmental data was not high enough to explain differences in rankings between field trials conducted at the same year–location combination. Similarly, we defined the sum of estimated variances associated with random effects for trial $\left( {zlv} \right)_{hjp}$ and trial:treatment interaction $\left( {zlvt} \right)_{hjpk}$ as unexplained environmental variances. Once again, the resolution of the environmental data was not high enough to explain differences between average trial performances from the same year and location.

#### Environmental covariates

Environmental covariates were aggregated to monthly mean values for all parameters (Table S3) starting 1st September of the sowing year and ending 31st August of the harvest year. In Central Europe, the sowing period for hybrid rye generally commences no earlier than mid to late September. Nevertheless, we wanted to make sure to also depict the sowing conditions that can have a strong effect on field emergence rates. Therefore, we decided to include weather data from the beginning of September onward. Ideally, weather data would have been obtained based on individual sowing and harvest dates of trials but those were not available to us for most trials. Furthermore, two physiological indices, namely the photothermal quotient and the heat–temperature sum were calculated for each month and environment. This amounts to a total number of 403 environmental covariates used as predictors in this study.

For the prediction in untested years, we used two different sets of weather data predictors in CV: 1) actual weather data from the year of interest and 2) weather data from all the remaining years in the training set (eleven years in total) as a proxy for the true weather data of an untested year. Our CV procedure assumes that all single years are independent random draws from the same distribution, and relatedness between years cannot be described by any autoregressive model.

#### Pre-processing pipeline

All numerical predictors as well as the target variable were transformed to zero mean and unit variance to equalize scales of parameters. The categorical predictors c*ountry, treatment, age of genotype* and *genotype* were translated into one-hot encoding. One-hot encoding corresponds to the concatenation of design matrices for each of the aforementioned categorical variables. Then, all predictors were brought into a single rectangular matrix of size *n* × *m*, where *n* corresponds to the number of unique yield observations and *m* corresponds to the number of predictor variables. Missing values in the categorical predictors were imputed by 1 divided by the number of unique levels of the respective predictor. This matrix was then used as input to the different ML and DNN models.

#### Target-variable engineering for ML and DNN models

Predicting environment-specific yield phenotypes aims to answer two distinct prediction problems at the same time: first, predicting the absolute yield productivity of an environment; and second, predicting the within-environment differences between genotypes or their rankings. Therefore, we decided to design a target variable that would allow a subsequent ML or DNN algorithm to focus only on the second prediction problem. We used the estimated coefficients from the random-effects model (1) described in Sect.  2.2.1. We defined two distinct target variables, which we termed yHatGE and yHatGGE, where yHatGE comprises the estimated GxE effects only and yHatGGE additionally comprises the best linear unbiased prediction (BLUP) of the genotype main effect:

2 $$\begin{gathered} yHatGE = \left( {\widehat{gt}} \right)_{ik} + \left( {\widehat{gz}} \right)_{ih} + \left( {\widehat{gl}} \right)_{ij} + \left( {\widehat{gzl}} \right)_{ihj} + \left( {\widehat{gzlt}} \right)_{ihjk} \hfill \\ yHatGGE = \hat{g}_{i} + yHatGE \hfill \\ \end{gathered}$$

The hat notation in Eq. (2) indicates that we used the empirical estimates, the BLUPs of the respective effects. Environmental main effects were excluded from both target variables since they do not affect the within-environment ranking of genotypes. In Fig. S3, the boxplots of the within-environment Spearman correlation coefficient (SCC) between yHatGGE and GDY show that the rankings were mostly conserved, with a mean SCC value of 0.94 for each CV scheme. However, there are also environments for which the correlations between the GDY-based rankings and the yHatGGE-based rankings were as low as ~ 0.2. This is because we excluded estimated effects from model (1) for the trial:genotype interaction $\left( {gzlv} \right)_{ihjp}$ and the residual ($\it e_{{{{inhjpk}}}}$) since their variance was not captured by the resolution of the environmental data as mentioned in Sect. 2.2. Hence, Fig. S3 shows the theoretical upper limit of predictability for GDY based on yHatG(G)E target variables. To avoid any information leakage and upward bias, the target variables were estimated separately for each CV split. Therefore, we fitted model (1) for each training set for all CV splits as outlined in Table 3.

**Table 3:** Cross-validation scheme overview

Scheme^a^	E	L	Y	LY
No of splits	139	32	9	139
Avg. training set size (%)	99.78	98.70	88.89	87.80

^a^ All schemes are leave-one-out schemes with unique location x year combinations (envCV; E), locations (L), years (Y) or one full year plus one full location (LY) being sequentially dropped from the training set

### Loss function engineering for ML and DNN models

The loss function defines the optimization objective of the user. During model training, the optimizer iteratively adjusts the model parameters to minimize the loss function, thereby improving the model’s predictive performance. Most regression problems strive to minimize the MSE of prediction, which is the average squared difference between the true and predicted values. This is the basis of least squares and the default loss function of common ML and DNN regression models (Abadi et al. 2016; Chen and Guestrin 2016; Ke et al. 2017).

**Average within-environment MSED**

We implemented MSED according to Piepho (1998) as custom loss function for all tested ML and DNN models. Environment, as defined in Sect. 2.1.2, was used as a grouping variable to calculate within-group means of prediction errors. Environment-wise MSED values following Eq. (3) were aggregated to an average score during optimization in each iteration of DNN model training.

3 $$\begin{gathered} MSED_{j} = \frac{{\mathop \sum \nolimits_{i = 1}^{{n_{j} }} \mathop \sum \nolimits_{{i^{\prime} \ne i}}^{{n_{j} }} \left( {y_{ij} - y_{{i^{\prime}j}} - \left( {z_{ij} - z_{{i^{\prime}j}} } \right)} \right)^{2} }}{{n_{j} \left( {n_{j} - 1} \right)}} = \frac{{2\mathop \sum \nolimits_{i = 1}^{{n_{j} }} \left( {f_{ij} - \overline{{f_{.j} }} } \right)^{2} }}{{\left( {n_{j} - 1} \right)}}, \hfill \\ \overline{MSED} = \frac{1}{J}\mathop \sum \limits_{j = 1}^{J} MSED_{j} \hfill \\ \end{gathered}$$

with $f_{ij} = y_{ij} - z_{ij}$, where *y* and *z* denote the observed and predicted values of the *i*-th genotype in the *j*-th environment, respectively. J and $n_{j}$ denote the total number of environments and the number of genotypes within environment j, respectively.

The ML models which we tested, namely XGBoost (XGB) from the scikit-learn Python library (Chen and Guestrin 2016) and LightGBM (LGBM) from the Microsoft Python library (Ke et al. 2017), optimize based on single predictions and not based on group statistics. Therefore, we reformulate the loss to:

4 $$L_{ij} = \left( {f_{ij} - \overline{{f_{.j} }} } \right)^{2}$$

XGB and LGBM expect loss functions to return both the gradient (first order derivative) and the diagonal of the Hessian (second-order derivative) of the loss function with respect to predictions $z_{ij}$. The returned gradient and diagonal of the Hessian are vectors of size $n = \mathop \sum \limits_{j = 1}^{J} n_{j}$.

5 $$\frac{{\partial L_{ij} }}{{\partial z_{ij} }} = - 2 \cdot \frac{{n_{j} - 1}}{{n_{j} }}\left( {f_{ij} - \overline{{f_{.j} }} } \right)$$

6 $$\frac{{\partial^{2} L_{ij} }}{{\partial z_{ij}^{2} }} = 2 \cdot \left( {\frac{{n_{j} - 1}}{{n_{j} }}} \right)^{2}$$

XGB and LGBM expect the losses of single prediction to be independent from each other with, i.e., $L = \mathop \sum \limits_{i} L_{i} \left( {z_{i} } \right)$. In that case the Hessian is a diagonal matrix since $\frac{\partial^{2} L}{\partial z_{i} \partial z_{i'}} = 0 \;\text{for } i \ne i'$ because losses of pairs of predictions do not interact. This is unrealistic for the introduced MSED loss function. The group-wise means $\overline{{f_{.j} }}$ introduce interdependence between the loss of pairs of predictions from the same environment. The full Hessian matrix can be calculated as follows:

7 $$\frac{{\partial^{2} L}}{{\partial z_{ij} \partial z_{i^\prime j^\prime } }} = \left\{ {\begin{array}{*{20}c} {2 \cdot \left( {\frac{{n_{j} - 1}}{{n_{j} }}} \right)^{2} \,\,\mathrm{if}\, i = i^\prime ,j = j^\prime } \\ { - 2 \cdot \frac{{n_{j} - 1}}{{n_{j}^{2} }} \mathrm{if}\, i \ne i^\prime ,j = j^\prime } \\ {0 \mathrm{if}\, j \ne j^\prime } \\ \end{array} } \right.$$

For pairs of predictions that belong to the same environment $j\left( i \right) = j\left( {i^\prime } \right)$ the off-diagonal elements are not zero since they interact via $f_{.j}$. Hence, the implementation we are proposing here is limited by the fact that XGB and LGBM do not offer to pass on the full Hessian for optimization but only its diagonal. The DNN was implemented via TensorFlow-Keras, which enables auto differentiation internally therefore the gradient and Hessian were not returned explicitly by the custom loss function. Due to the unbalanced nature of the motivating dataset, within-environment metrics were calculated first since the number of genotypes varies between environments.

Linear mixed-model optimization is classically based on residual maximum likelihood (REML), which strives to maximize the likelihood of the response variable vector *y* with regards to the variance components of the different model terms. Therefore, there is no need to adapt the optimization procedure of LMMs to focus on GxE prediction using custom loss functions.

### Models

To test the effectiveness of our designed GxE target variable and the custom GxE loss function, we implemented two different ML models and one DNN model, which will be described in the following section. Furthermore, we adapted a parametric linear mixed model including environmental covariates in form of an environmental kinship matrix adapted from Jarquín et al. (2014), which we will call the E-BLUP model. We used this model to benchmark the performances of the ML and DNN models. Due to the lack of marker data and since our MET dataset covers more than 10 years of OVT data, we decided to include the age of genotypes as categorical explanatory variable to allow the models to separate the general genetic gain from the remaining effects.

### E-BLUP

As explained above, the E-BLUP model was used as a baseline approach in this study. Based on the environmental covariates for weather and for soil described in 2.2.2, a linear environmental kinship matrix was created following Jarquín et al. (2014):

8 $$E = \frac{{EC*EC^{T} }}{{mean\left( {diagonal\left( {EC*EC^{T} } \right)} \right)}}$$

where EC is the centered and scaled (z-transformed) matrix of environmental covariates, and $EC^{T}$ is the transpose of the EC matrix. The *E* matrix is a proxy for the kinship between respective environments. Furthermore, we provided the model with the following factor inputs as fixed effects: (1) the country and (2) the treatment (intensive or extensive) as well as (3) the first year of national list testing of genotype *i* as proxy for the age of a genotype.

We used the model

9 $$M_{injk }^{\left( 1 \right)} = \mu + c_{n} + t_{k} + a_{r} + g_{i} + w_{j} + gw_{ij} + \varepsilon_{injk}$$

where μ is the fixed intercept, $c_{n}$ is the fixed country effects, $t_{k}$ is the fixed treatment effect, $a_{r}$ is the fixed genotype-age effect, $g_{i}$ is the random independent and identically distributed (IID) genotype effect with $g_{i} \sim N\left( {0,\sigma_{g}^{2} } \right)$, $w_{j}$ is the random envCV effect, $gw_{ij}$ the random interaction effect between genotype and envCV and $\varepsilon_{injk}$ is the random error term that is assumed to be IID with constant variance $\varepsilon_{injk} \sim N\left( {0,\sigma_{\varepsilon }^{2} } \right)$. The vector *w* is modeled as $w \sim N\left( {0,\left[ {Z_{w} EZ_{w}^{\prime } } \right]\sigma_{w}^{2} } \right)$, where *E* is the environmental relatedness matrix described in Eq. (4). The GxE interaction term is modeled as $gw \sim N\left( {0,\left[ {Z_{g} Z_{g}^{\prime } } \right] \circ \left[ {Z_{w} EZ_{w}^{\prime } } \right]\sigma_{gw}^{2} } \right)$ where $Z_{g}$ is the genotype incidence matrix, *E* is the environmental kinship matrix as defined in Eq. (4), $Z_{w}$ is the envCV incidence matrix and ∘ denotes the Hadamard product between two matrices. Different to Jarquín et al. (2014), no genomic relationship matrix was created since we did not have any genetic marker information available for the dataset at hand.

The linear mixed model from Eq. (5) was fitted using the R package BGLR: Bayesian Generalized Linear Regression (Perez and de los Campos 2014). The random effects for *g*, *w* and their interaction *gw* were implemented using kernel matrices derived from eigendecomposition and fitted via reproducing kernel Hilbert space models. Specifically, as inputs to the BGLR algorithm we used the eigenvectors matrix (*V*) and eigenvalue vector (*d*) of matrices w and gw as described in Perez and de los Campos (2014). We ran BGLRs internal sampler, MCMC for 2500 iterations and the first 500 iterations were removed as burn-in with thinning equal to 5.

#### Machine-learning models

We implemented two different gradient boosting algorithms (GBA) in this paper: XGB and LGBM. The foundation for GBAs was laid by Friedman (2001). GBAs are ensembles of sequentially fitted decision trees. Each new tree is trained on the residuals of the previous one. The final model is essentially a weighted sum of all the individual models. All hyperparameters were tuned using Optuna Python library (Akiba et al. 2019). Detailed information on the parameters tuned and the respective spaces sampled from can be found in Table S2.

#### Deep neural network

A DNN was implemented to predict within-environment-specific genotype performance differences using TensorFlow-Keras Python library (Abadi et al. 2016). The DNN consisted of five dense layers with drop-out layers between dense layers 1 and 2 as well as 2 and 3 drop-out layers. Various measures were taken to minimize overfitting: (1) Drop-out layers were included in the DNN architecture. (2) L2 regularization was used in every dense layer to handle multicollinearity and reduce overfitting. (3) A custom callback function was created making sure in each epoch that new model weights were only saved when the validation loss was reduced, and the training loss was maximum 50% smaller than the validation loss. Furthermore, we used the ratio between training loss and validation loss from the first epoch as scaling factor inside the callback to set training and validation loss to the same start levels and allow the callback to focus on a similar decay rate for both training and validation loss. This makes sure the trained model generalizes well to the validation set. (4) The validation set was designed to mimic the CV strategy. We performed a semi-random 90/10 split into training set and validation set. About 90% of the validation set was comprised of locations that were not present in the training set. These locations were selected using stratified sampling based on k-means clustering on the environmental data of the training set. Using the elbow method, we determined 10 clusters of envCVs. Then, we randomly sampled complete locations until the validation set contained 100% cluster coverage and maximum deviation of 10% from the target size. The remaining ~ 10% of the validation set were randomly sampled envCVs from the training set.

For the final prediction we used an ensemble model of ten DNNs that differed only in the random weights used for initialization to improve model robustness and handle the variance-bias trade-off, as described by Kick and Washburn (2023). The ensemble prediction is a simple average obtained from the ten individual DNN model predictions. All hyperparameters were tuned using Optuna Python library (Akiba et al. 2019). Detailed information can be obtained from Table S2. All DNN trainings and predictions were GPU-accelerated on NVIDIA GeForce RTX 3050.

Additionally, in DNNs with MSED-loss optimization, we implemented group-aware batching using environment as grouping variable to make sure each batch would contain complete environments. This approach is necessary because the MSED calculation requires group statistics (Sect. 2.4), which can become biased or fail entirely if batches contain incomplete environments with very few observations.

### Cross-validation

We provide comprehensive results on four CV schemes, namely leave-one-envCV-out (E), leave-one-location-out (L), leave-one-year-out (Y) and leave-one-year-and-one-location-out (LY). The difficulty of the prediction problems increases in the following order: E, L, Y and LY. Every split was sampled from the VP trial network that was used as test set since it has the most consistent set of genotypes across years and locations (Fig. S2) and the highest genotype connectivity to other trial networks (Fig. S1b) across a large region (DE and PL) and time span (2016 to 2024). Consequently, this approach has the advantage that variations in predictive performance between environments are primarily attributed to genuine environmental differences, rather than to changes in genotype composition or suboptimal connectivity. The E CV splits were based on envCV; hence, both treatment levels were excluded in the same split. Additionally, “location” does not refer to a unique geographic coordinate, but rather to the designation of a trialing location, where spatially proximate fields used across years are considered part of the same location. Hence, in L and LY we made sure to exclude any trials that were associated with the location of the respective split. As mentioned earlier, some trial networks have overlapping envCVs or locations. In that case, we also made sure to exclude those from the training set in the respective CV split even though only VP data were used later to calculate CV metrics. The same is evidently true also for Y and LY, where the complete year across trial networks was excluded from the training set but only the VP trial network data were then used as test set to generate CV metrics.

Both Y and LY are relatively costly CV strategies with regard to the reduction in training set size. However, since the number of years in our test set is relatively large, the average reduction in training set size of approximately 12% for Y and LY is rather small. Therefore, we expect a potential negative effect on accuracy in Y and LY to be neglectable.

### Metrics and benchmarks

#### Metrics

We used the within-environment Pearson correlation coefficient (PCC) as evaluation metric. We also included the within-environment SCC. Furthermore, we used the within-environment MSED, according to Piepho (1998) and as described in Sect. 2.4, not only as loss function but also as CV metric.

**Average within-environment rooted MSED**

Calculation was performed according to Sect. 2.4, Eq. (3). Before averaging across environments, square roots of group-wise MSED values were calculated in order to re-scale to the original target variable.

**Average within-environment PCC**

The average environment-wise PCC $\overline{r}$ is computed by first calculating the PCC within each environment ($r_{j}$) and then averaging these values across all J environments. Given a dataset divided into J environments, where each environment *j* contains pairs of observed and predicted data points $\left( {y_{ij} ,z_{ij} } \right)$ for each genotype *i,* the formula for the within-environment PCC is:

10 $$r_{j} = \frac{{\mathop \sum \nolimits_{i = 1}^{{n_{j} }} \left( {y_{ij} - \overline{y}_{.j} } \right)\left( {z_{ij} - \overline{z}_{.j} } \right)}}{{\sqrt {\mathop \sum \nolimits_{i = 1}^{{n_{j} }} \left( {y_{ij} - \overline{y}_{.j} } \right)^{2} } \sqrt {\mathop \sum \nolimits_{i = 1}^{{n_{j} }} \left( {z_{ij} - \overline{z}_{.j} } \right)^{2} } }},$$

The average within-environment PCC is then calculated as follows:

11 $$\overline{r} = \frac{1}{J}\mathop \sum \limits_{j = 1}^{J} r_{j}$$

**Average within-environment SCC**

True ranks and predicted ranks within environments were calculated. Those were used as inputs to Eqs. (10) and (11) to compute the SCC. This metric is less prone to bias, e.g., by genotypes that tend to always perform much lower than others and hence are easy to predict and have a strong effect on PCC due to their large difference to the average. This can lead to an upward bias in PCC, which is avoided by the SCC.

#### Benchmarking against the genotypic main effect

In the 2023-G2F challenges, three of the highest-ranked groups (based on within-environment PCC values), did not include any environmental or management data into their models but only genomic markers and experimental design factors (Washburn et al. 2024). In effect, these groups implemented classical marker-based genomic selection models, although the initiative’s aim was to incentivize the development of GxE predictive models. This highlights the importance of benchmarking GxE-prediction approaches against purely phenotype- or genetics-driven baseline models like classical (G)BLUP (Malosetti et al. 2016).

Therefore, we benchmarked model performances against the predictive power of the simple BLUPs of the genotype main effects $\hat{g}_{i}$ from the random effect model in Eq. (1), which was fitted in each CV split. Likewise, in the absence of phenotypic data, genomic estimated breeding values (GEBVs) can serve as a benchmark for evaluating the outcomes of GxE models. This benchmark allowed us to evaluate if the proposed models successfully capture the proportion of the GxE variance in the data, or if it would have been preferable to assume stable genotype rankings across environments based on $\hat{g}_{i}$ or GEBVs.

### Software and implementation

Target variable estimation in ASReml (Butler DG 2023) was developed in R. The LMM-based E-GBLUP model was as well written in R using the BGLR package from Perez and de los Campos (2014). Python codes were written for ML and DNN models and any associated methodology we have described in this paper. Codes are available on GitHub under https://github.com/weramaria/rye_gxe_prediction.git. Auxiliary package code used in the repository can be found at https://github.com/weramaria/utils_package.git. We limited ourselves to those functions that are likely to be of interest to readers working with similar datasets.

## Results

In this study we are demonstrating two different ways of guiding ML and DNN models to focus on predicting pure genotype effects and GxE-associated effects only. As was explained in the Methods, this is done by a) designing GxE target variables and b) designing a loss function, which optimizes the within-environment pairwise genotype differences. In the following sections, we will stick to the nomenclature summarized in Table 4.

**Table 4:** Results nomenclature overview

Label	Target variable used in training	Loss function used in training
GDY	GDY	MSE
yHatGE + $\hat{g}_{i}$	yHatGE	MSE
yHatGGE	yHatGGE	MSE
GDY MSED	GDY	MSED

### Sources of variance in European MET rye dataset

**Fig. 2:**
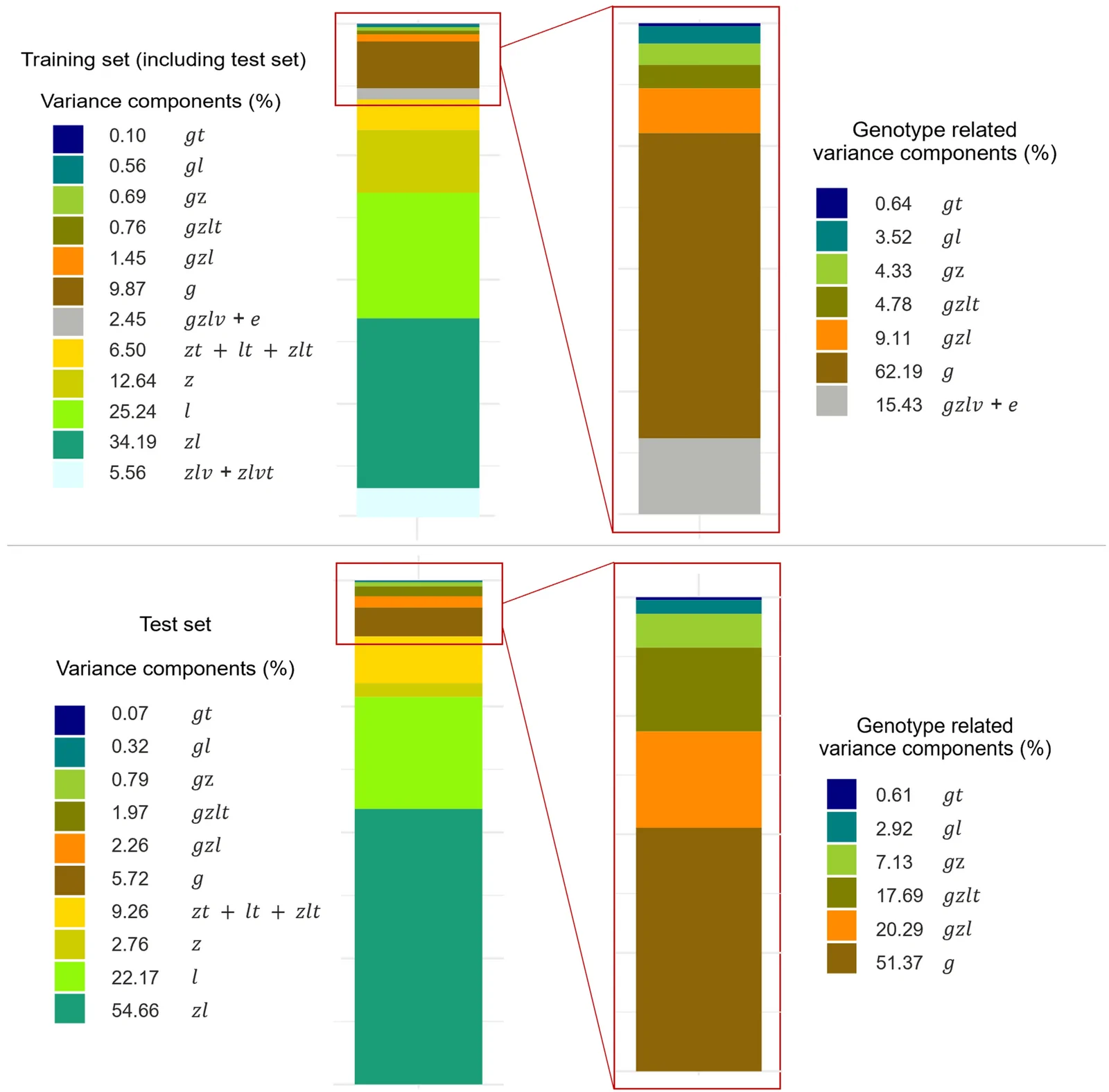
Relative contributions (%) of the estimated variance components from the mixed model in Eq. (1) for the training set including the test set (top) and for the test set alone (bottom). Left stacked bars show the full variance decomposition; right stacked bars show genotype-related variance components only, rescaled to 100% within that subset. Notation follows Eq. (1): z, year, l, location and zl, year × location interaction. Grouped terms zt+lt+zlt denote the estimated relative interaction variances of the latter three components with treatment (t). The main genotype variance component is denoted as g. Furthermore, gt, gl, gz, gzl, and gzlt denote genotype-related interaction variance components. The grouped term zlv+zlvt denotes the variance components of trial nested within year × location, and of its interaction with treatment which are treated as unexplained environmental variances. Similarly, gzlv+e for the genotype × nested-trial variance component combined with residual error variance are treated as unexplained genotype-related variances. Both grouped terms cannot be explained by the environmental data available in this study due to limited spatial resolution

Figure 2 shows the results of variance decomposition based on the LMM presented in Eq. (1). Most of the variance in the motivating rye dataset is explained by main environmental factors attributed to terms $z_{h} ,l_{j} ,\left( {zl} \right)_{hj}$ and their interactions with treatment as defined in Eq. (1). These terms together explain 84.1% of the total variance in the training set and 88.8% of the total variance in the test set. Since our rye MET dataset spans comparably large dimensions both in space (five EU countries) and time (years 2013 to 2024), this was expected (Laidig et al. 2017; Kick and Washburn 2023; Avagyan et al. 2025). The remaining ~ 15% (training set) to 11% (test set) of the total variance are associated with the genotypes and their interactions with the environmental factors. This result underlines the importance of techniques that enable ML and DNN algorithms to dissect genotype-associated effects from environmental main effects. The variance associated with the genotype main effects is approximately of the same magnitude as the sum of the GxE interaction variances, with about 40% GxE variance in the training set and about 50% in the test set out of the total genotype-related variances in each set (Fig. 2). About 15% of the genotype-associated variances are explained by the interaction between trial and genotype, $\left( {gzlv} \right)_{ihjp}$, as well as the three-way interaction between trial, treatment and genotype, which is absorbed by the residual error variance of the model presented in Eq. (1). As explained in Sect.  2.2.1, we defined these two variances as “unexplained,” since the resolution of environmental data is too low to explain this variation.

### Designing GxE target variables to guide ML and DNN models

**Fig. 3:**
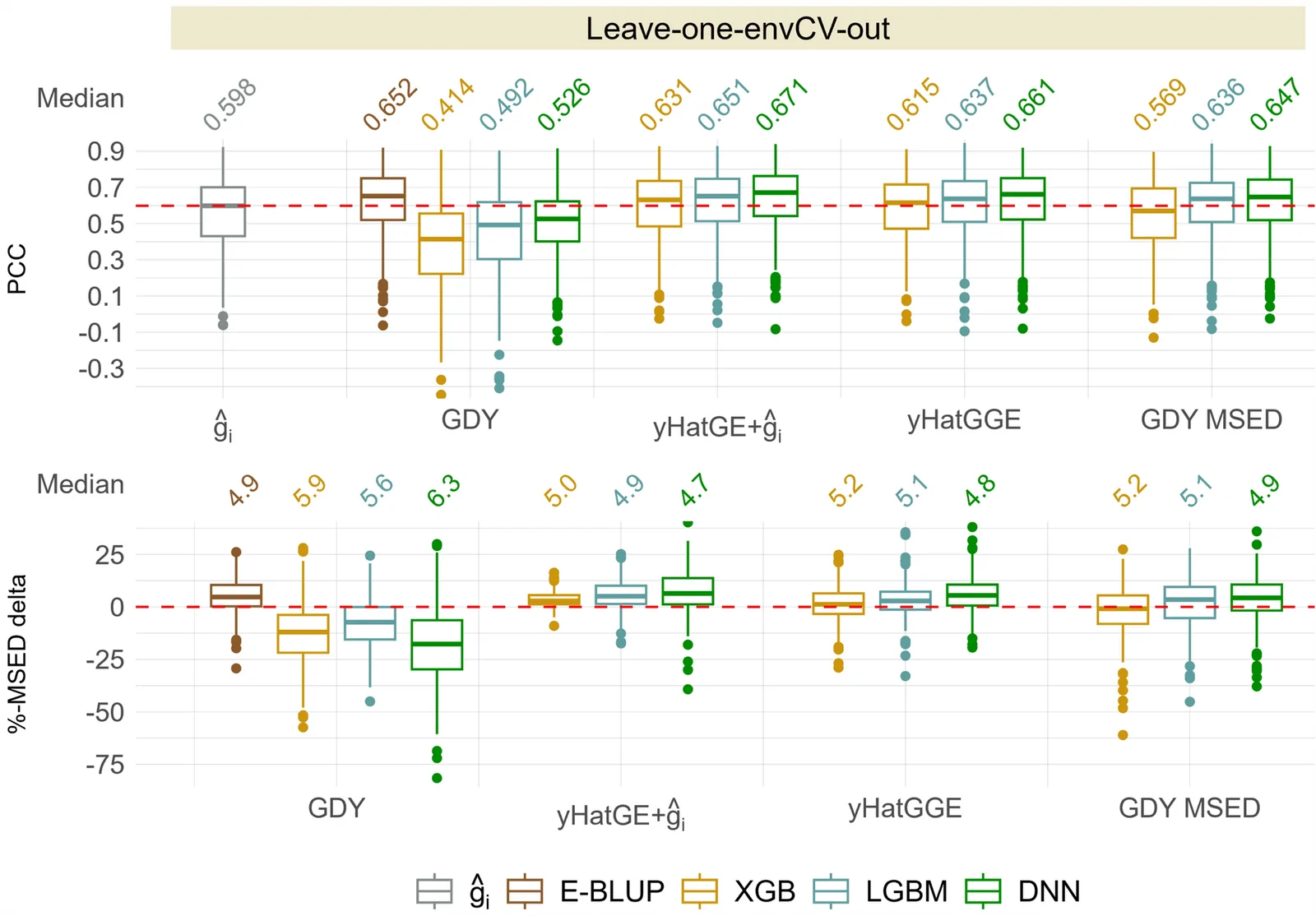
Results from leave-one-envCV-out cross-validation, where envCV represents a unique location x year combination. The accuracy is measured as within-environment Pearson correlation coefficient (PCC) and percentage decrease of within-environment mean squared error of differences (MSED) of tested approaches over $\hat{g}_{i}$, the genotype main effect estimated as best linear unbiased prediction (BLUP). Four different models in combination with three different target variables and a MSED-loss-function approach were compared. The models included an environmental-kinship-based BLUP model adapted from Jarquín et al. (2014) (E-BLUP), two extreme gradient boosting algorithms XGBoost (XGB) and LightGBM (LGBM), and a deep neural network (DNN). The target variables included grain dry matter yield (GDY) and two constructed target variables, specifically yHatGE and yHatGGE, which are defined as linear combinations of estimated coefficients derived from the variance components decomposition of GDY. After prediction, $\hat{g}_{i}$ was added to yHatGE. The dashed red line represents the median value of the respective metric for the lower benchmark, $\hat{g}_{i}$

**Fig. 4:**
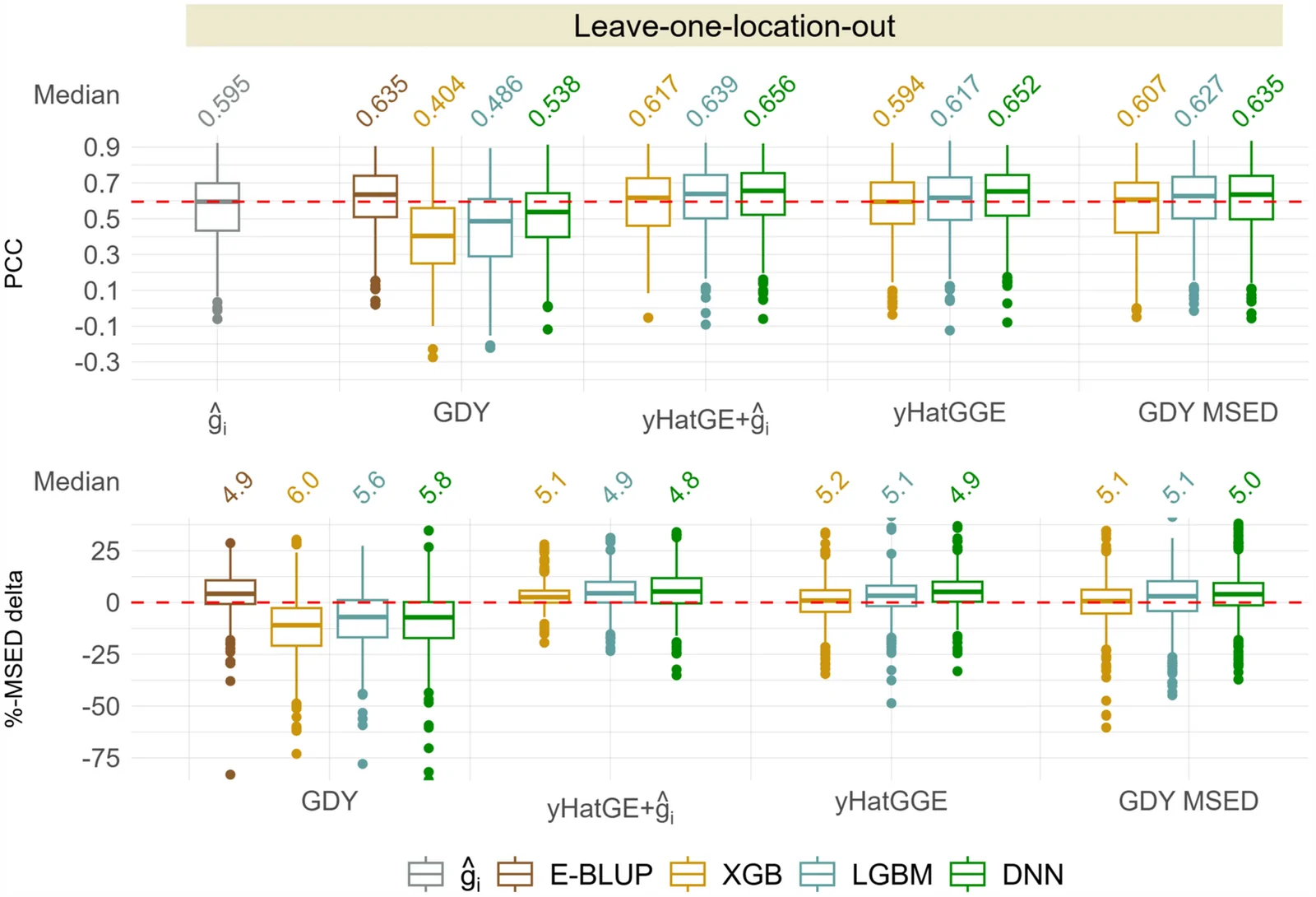
Leave-one-location-out cross-validation results measured as within-environment Pearson correlation coefficient (PCC) and percentage decrease of within-environment mean squared error of differences (MSED) of tested approaches over $\hat{g}_{i}$, the genotype main effect estimated as best linear unbiased prediction (BLUP). Four different models in combination with three different target variables and a MSED-loss-function approach were compared. The models included an environmental-kinship-based BLUP model adapted from Jarquín et al. (2014) (E-BLUP), two extreme gradient boosting algorithms XGBoost (XGB) and LightGBM (LGBM), and a deep neural network (DNN). The target variables included grain dry matter yield (GDY) and two constructed target variables, specifically yHatGE and yHatGGE, which are defined as linear combinations of estimated coefficients derived from the variance components decomposition of GDY. After prediction, $\hat{g}_{i}$ was added to yHatGE. The dashed red line represents the median value of the respective metric for the lower benchmark, $\hat{g}_{i}$

**Fig. 5:**
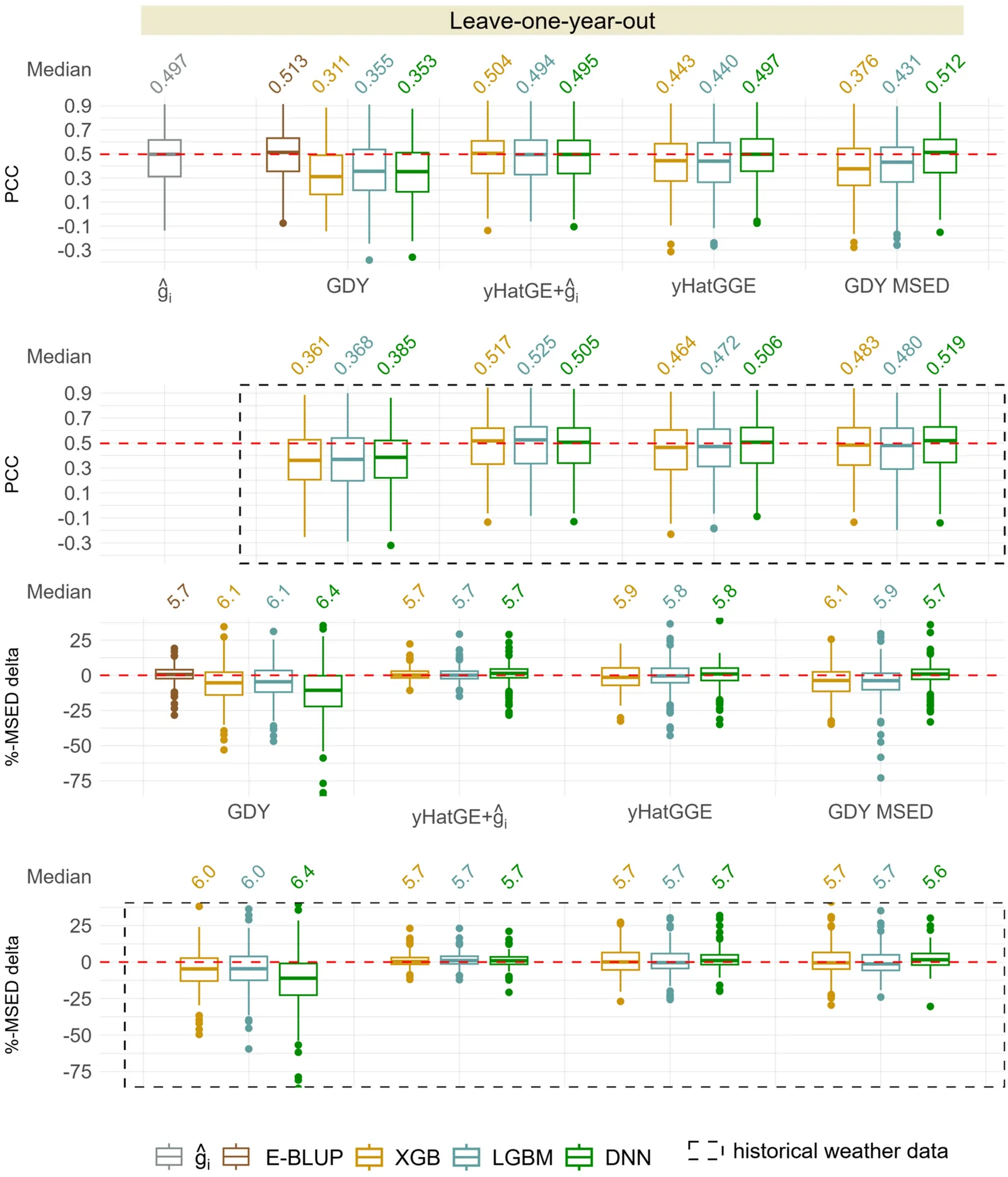
Leave-one-year-out cross-validation results measured as within-environment Pearson correlation coefficient (PCC) and percentage decrease of within-environment mean squared error of differences (MSED) of tested approaches over $\hat{g}_{i}$, the genotype main effect estimated as best linear unbiased prediction (BLUP). Four different models in combination with three different target variables and a MSED-loss-function approach were compared. The models included an environmental-kinship-based BLUP model adapted from Jarquín et al. (2014) (E-BLUP), two extreme gradient boosting algorithms XGBoost (XGB) and LightGBM (LGBM), and a deep neural network (DNN). The target variables included grain dry matter yield (GDY) and two constructed target variables, specifically yHatGE and yHatGGE, which are defined as linear combinations of estimated coefficients derived from the variance components decomposition of GDY. After prediction, $\hat{g}_{i}$ was added to yHatGE. Labels positioned between plots of the same metric consistently apply to both the upper and lower rows of plots. The dashed red line represents the median value of the respective metric for the lower benchmark, $\hat{g}_{i}$. The results shown within the dashed rectangle are derived from median predictions utilizing twelve years of historical weather data

**Fig. 6:**
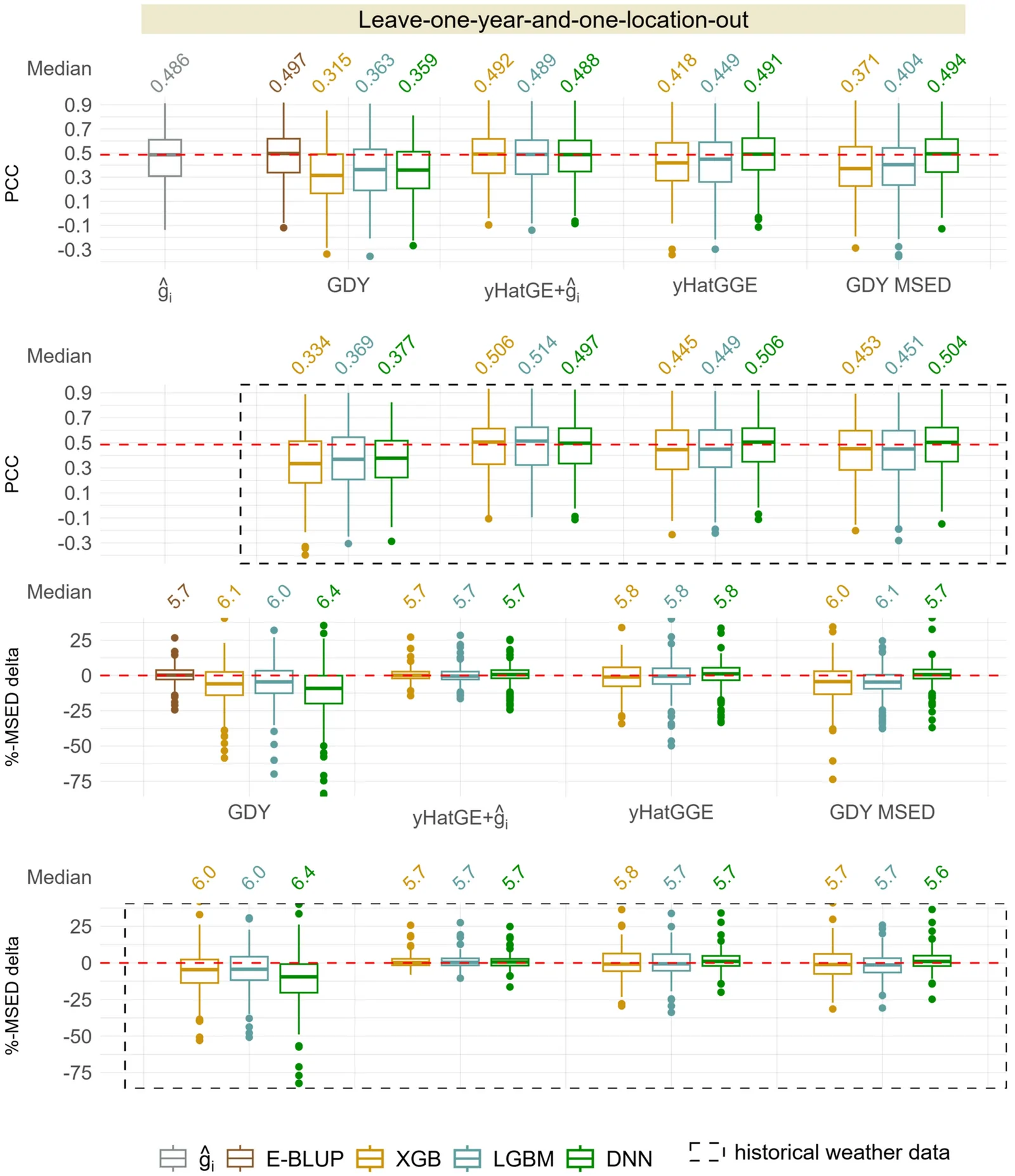
Leave-one-year-and-one-location-out cross-validation results measured as within-environment Pearson correlation coefficient (PCC) and percentage decrease of within-environment mean squared error of differences (MSED) of tested approaches over $\hat{g}_{i}$, the genotype main effect estimated as best linear unbiased prediction (BLUP). Four different models in combination with three different target variables and a MSED-loss-function approach were compared. The models included an environmental-kinship-based BLUP model adapted from Jarquín et al. (2014) (E-BLUP), two extreme gradient boosting algorithms XGBoost (XGB) and LightGBM (LGBM), and a deep neural network (DNN). The target variables included grain dry matter yield (GDY) and two constructed target variables, specifically yHatGE and yHatGGE, which are defined as linear combinations of estimated coefficients derived from the variance components decomposition of GDY. After prediction, $\hat{g}_{i}$ was added to yHatGE. Labels positioned between plots of the same metric consistently apply to both the upper and lower rows of plots. The dashed red line represents the median value of the respective metric for the lower benchmark, $\hat{g}_{i}$. The results shown within the dashed rectangle are derived from median predictions utilizing twelve years of historical weather data

Predictions based on target variables yHatGE and yHatGGE differ only in the sequence in which the estimated genetic main effect $\hat{g}_{i}$ was incorporated into the ML or DNN prediction. Because of how yHatGE was built, models based on that variable were trained to predict the GxE effects only. Afterward, we added the estimated genetic main effect coefficients of each respective CV split to the yHatGE-based predictions from the ML/DNN model. Results based on this combination will have the label yHatGE + $\hat{g}_{i}$ throughout this study (Table 4). yHatGGE instead is the sum of both the estimated GxE effects and $\hat{g}_{i}$. Hence, models based on yHatGGE were trained to predict both the GxE effects and the genotype main effects. The results of all four CV schemes, across evaluation metrics, showed that predictive abilities for environment-specific genotype performances were significantly lower with GDY as the target variable than with the designed target variables yHatGE and yHatGGE (Figs. 3–6). This was the case for all ML and DNN models we tested. The greatest improvement across CV schemes we found for XGB based on yHatGE + $\hat{g}_{i}$, ranging between + 62 to + 52% increase in median PCC. Also, for LGBM we observed that predictions from yHatGE + $\hat{g}_{i}$ were consistently superior with an increase in median PCC over GDY between + 31.5% and + 39%. For DNNs, improvement levels varied stronger between CV schemes, ranging from + 21% for L (Fig. 4) and + 42% for Y (Fig. 5). Generally, the difference between predictions based on both target variables was small for DNN models, although yHatGGE was superior to yHatGE in Y/LY (Fig. 6) and vice versa for E/L (Fig. 4). The difference between approaches yHatGE + $\hat{g}_{i}$ and yHatGGE was small across models for E and L (Figs. 3, 4). Only in the Y and LY CV schemes for XGB and LGBM yHatGE + $\hat{g}_{i}$ showed a clear advantage (Figs. 5, 6). In contrast, for DNNs, yHatGGE continued to yield better predictive performances across CV schemes.

### Designing GxE loss functions to guide DNN and ML

To overcome the dominance of environmental main effects in GxE prediction, another alternative strategy next to GxE target-variable engineering is the formulation of GxE loss functions. All three Python libraries that we tested XGBoost, LightGBM and TensorFlow-Keras, allow users to implement custom loss functions.

As has been described in the Materials and Methods section, we identified the MSED as a suitable loss function since it optimizes based on the residuals of predicted pairwise genotype differences within environments and thereby factors out environmental main effects.

We could show that predictive ability expressed as median PCC for the MSED-optimized DNNs was almost identical to median PCC of the DNNs trained on the GxE target variables yHatG(G)E across CV schemes and therefore effectively outperformed modeling strategies based on GDY in combination with MSE loss functions (Figs. 3–6). While for E and L (Figs. 3, 4), we found that GDY MSED delivered slightly weaker predictions than the yHatG(GE) counterpart models (median percentage-MSED delta 0.9% to 2.2% smaller), for Y and LY we found that the GDY MSED-based results even reached the highest accuracy of all tested approaches with an increase over $\hat{g}_{i}$ in median SCC of 7.9% and 6.6%, respectively (Table S6). We obtained very promising results for DNNs using MSED loss, but the picture was less clear for XGB and LGBM. Model performances for LGBM and XGB fell behind those of yHatG(G)E especially in Y and LY CV. Notably this deficit was almost fully recovered when the prediction was made based on historical weather records (Figs. 5, 6).

Overall, using a MSED-based custom loss function in the ML and DNN models substantially increased predictive ability compared to conventional ML and DNN models defaulting to MSE or MAE loss by 23%, 18%, 45% and 37% median PCC increase in E, L, Y and LY, respectively. Furthermore, the MSED-loss-function approach convinces due to its simplicity compared to the two-stage target-variable-design approach. Our results indicate that when applied in combination with DNN models, the accuracy is only slightly smaller relative to the designed target variables.

### Realized gain of presented methodology

**Genetic main effect as benchmark**

To be able to evaluate if the tested GxE-prediction approaches actually generated added value, we benchmarked them against $\hat{g}_{i}$—the estimated genetic main effect BLUPs from each respective CV split. Using $\hat{g}_{i}$ as prediction for the environment-specific genotype performances yielded median PCC values of 0.598, 0.594, 0.497 and 0.486 (Figs. 3–6) and median rooted MSED values of 5.2, 5.3, 5.7 and 5.7 dt ha^−1^ for E, L, Y and LY, respectively. Only models which outperformed this set of metrics did usefully exploit GxE and thereby created benefit. Generally, we observed that model improvements over $\hat{g}_{i}$ for any tested approach in Y and LY (Fig. 5) were smaller than in E and L CV (Figs. 3, 4).

As described earlier, classical GDY-based ML and DNN models with default MSE/MAE loss function showed the lowest performance across CV schemes and models and more importantly, they considerably underperformed $\hat{g}_{i}$, with reductions in median PCC ranging from -9.4 to -37%. This means, a user interested in environment-specific genotype predictions would achieve more reliable results by relying solely on $\hat{g}_{i}$ for decision-making. Overall, these results underline the importance of carefully selecting relevant benchmarks, such as $\hat{g}_{i}$, to avoid information loss or even biased conclusions.

In contrast, all ML and DNN model predictions based on GxE targets yHatGE and yHatGGE, as well as MSED loss outperformed $\hat{g}_{i}$ with respect to all three metrics in CV schemes E and L. The greatest enhancements were as high as + 15% in median SCC compared to $\hat{g}_{i}$ in E and + 13.7% in L CV using the DNN model trained on yHatGE + $\hat{g}_{i}$ and yHatGGE, respectively (Table S6). For Y and LY, only DNNs were able to consistently show slightly higher performances than $\hat{g}_{i}$. On the other hand, XGB and LGBM were especially losing predictive power when used in conjunction with MSED. Predictions based on yHatGE + $\hat{g}_{i}$ across models showed the same or slightly improved performances over $\hat{g}_{i}$ which is likely because, especially in Y and LY, yHatGE + $\hat{g}_{i}$ predictions were strongly approximating $\hat{g}_{i}$ values with median PCC between $\hat{g}_{i}$ and GxE predictions between 0.983 and 0.995 (Fig. S5). Especially under Y and LY this was more pronounced for yHatGE + $\hat{g}_{i}$ than for any other approach (Fig. S5).

**Environmental kinship LMM as benchmark**

As explained in 2.5.1, we adapted the E-BLUP model suggested by Jarquín et al. (2014) as a benchmark for our tested ML and DNN models. Our results showed that E-BLUP remained competitive and consistently outperformed XGB and LGBM in almost all cases with respect to metrics PCC (Figs. 3–6) and SCC (Table S6). Differences between DNNs and E-BLUP were small across CV-target-loss combinations ranging between + 3.3 to −3.5 median PCC (Figs. 3–6). Still, DNNs showed slightly increased performance over E-BLUP for at least one of the novel engineering approaches in each CV scheme apart from Y. Furthermore, when looking at median percentage-increase of rooted MSED over $\hat{g}_{i}$, we saw that although E-BLUP performed similar to LGBM and DNN approaches in CV scheme E (Fig. 3), it lagged behind, especially DNNs' performance in all other CV schemes. Generally, for Y (Fig. 5) and LY (Fig. 6), the improvement in MSED compared to $\hat{g}_{i}$ was, if at all, only very slight for any of the tested approaches. Again, only DNNs showed small positive improvements by using the MSED loss function in almost all cases. Overall, this study indicates, that guiding DNNs to focus on G × E-relevant data patterns—through target-variable or loss-function engineering—can outperform or at least match classical parametric modeling approaches. At the same time, it offers significant advantages in computing time and resource requirements compared to the classical E-BLUP model (Table 5). For E-BLUP, constructing the necessary kernels alone required approximately 34 h, and fitting a single model took about 12 times longer than for a DNN, and roughly 120 and 470 times longer than for XGB and LGBM, respectively.

**Table 5:** Overview of computing times and required computational resources for the different models

Model^1^	Pre-processing time (sec)	Model run time (sec)	RAM (GB)	GPU memory (GB)
E-BLUP	122,400.00	4680.00	~ 70	-
XGB	59.49	38.67	~ 12	-
LGBM	59.49	10.21	~ 12	-
DNN	59.49	381.70	~ 13	2.8

^a^ E-BLUP: Environmental-kinship-based linear mixed model, XGB: XGBoost, LGBM: LightGBM, DNN: deep neural network

Across methods, we observed substantial variability in the predictive performance between environments, with PCC values ranging from −0.08 to 0.94 for E and L (Figs. 3, 4) and from −0.32 to 0.94 for Y and LY (Figs. 5, 6) Nevertheless, compared to $\hat{g}_{i}$, the interquartile range (IQR) of predictive abilities was reduced by 20%, 14%, 11% and 15% in E, L, Y and LY, respectively. yHatG(G)E-based DNN predictions showed the smallest IQR of PCCs across most CV schemes and were consistently lower than IQRs observed for E-BLUP predictions. Despite these enhancements, certain individual environments continued to exhibit very low predictive ability across all evaluated approaches (Figs. 3–6).

### Inference for future years

We examined the use of historical weather data as a proxy for the likely conditions at a given site in the future. Our results for Y and LY CV indicate that predictions based on historical weather records for untested years can give more accurate estimates of per-environment genotype rankings than $\hat{g}_{i}$. The highest median improvement in SCC compared to $\hat{g}_{i}$ using historical weather data was found for yHatGGE DNN and GDY MSED DNN (+ 9.8%) in Y and for yHatGGE DNN (+ 9.9%) in LY (Table S6). It should be noted that all years for which we obtained historical weather records were present in the training dataset. Overall, our results suggest that the use of multiple years of historical weather data is a viable option to infer site-specific genotype rankings for a given year in the future.

Furthermore, when comparing predictions within the same target/loss-model approach, we observed that historical weather data-based predictions surprisingly slightly outperformed predictions based on the actual weather data of the year of interest across metrics and CV schemes. The advantage was mostly small yet consistent with a median increase in accuracy across tested approaches of ~ 5%. Since all approaches showed only very limited ability to infer accurate predictions from weather data of untested years, this suggests new year’s sets of weather data deviated substantially from the past and borrowing strength from related parameter combinations is difficult. This is supported by the distribution of environment relatedness, which is substantially higher for environments from the same year across the training set (median correlation 0.4) than for environments from distinct years (median correlation −0.1) (Fig. S4). Generally, we observed that the correlation between $\hat{g}_{i}$ and our predictions increased with growing complexity of the CV scheme (Fig. S5), underlining the difficulty to predict GxE effects compared to genetic main effects. For E and L, predictions based on historical weather data are irrelevant since, per definition, both CV schemes reflect scenarios in which predictions are made for genotypes in already tested years and were therefore not created. Overall, our results suggest that historical weather data improve inference for genotype performances in untested years.

## Discussion

### Disentangling trait prediction problems and turning them into GxE-prediction problems

Prior studies have shown that the predictive performance of ML and DNN models often falls behind parametric approaches based on linear mixed models and Bayesian theory that explicitly model GxE interactions in their model terms (Ray et al. 2023; Barreto et al. 2024). In this study, we present two novel approaches for improving the accuracy of GxE-prediction ML and DNN models and demonstrate their effectiveness on a representative hybrid rye dataset from Central Europe. Our developed methodology on (*1) target-variable engineering* and (*2) loss function engineering* significantly extends the existing toolbox of the field. Both approaches arise from the idea that breeders are primarily interested in understanding the ranking of genotypes at a given site, whereas the average productivity of that site is of minor importance. Accordingly, we partitioned the genotype yield prediction problem into its three components: (1) environmental main effect prediction, (2) genotype main effect prediction and (3) GxE interaction effect prediction. While classical mixed-model or Bayesian approaches inherently allow for this dissection via the formulation of dedicated model terms, this is not possible in common ML or DNN algorithms. Therefore, the proposed methodology effectively contributes to overcoming this limitation by allowing ML and DNN models to focus only on components (2) and (3) of the yield prediction problem.

Khaki and Wang (2019) was one of the first publications using DNNs in the GxE modeling context but in contrast to this study, did focus on predicting absolute yield rather than environment-specific genotype differences or rankings. More recently Jubair et al. (2023), Kick et al. (2023) as well as different teams of the Genomes2Fields (G2F) challenge (Washburn et al. 2024) used DNNs for predictive GxE modeling in plant breeding. With the target variable engineering approach, we took advantage of LMMs capability to dissect response variables into dedicated effect coefficients. We can find parallels between the yHatGE + $\hat{g}_{i}$ and yHatGGE prediction approach and (weighted) average-based ensemble model approaches of classical parametric BLUP-based methods with ML and deep-learning models, which can have greater predictive power than any of the two approaches standalone (Kick and Washburn 2023). We can also find similarity between our approach and the idea of the 2018 Syngenta Crop Challenge of providing the yield difference between individual corn hybrids and the average yield of an environment as feature variable to the participants. However, similar to using the environment-wise relative yields of genotypes, this is problematic in unbalanced datasets where differences in average yields are not solely attributed to environmental factors but also to the changing sets of genotypes across environments. Khaki and Wang (2019) aimed to predict the yield difference in the 2018 Syngenta Crop Challenge but concluded that models predicting genotype yield directly are more accurate presumably since they measured model accuracy as across-environment rooted MSE and PCC, which inherently favor the prediction of environmental main effects over genotype rankings. Another related line of research is presented in the recent approach proposed by Avagyan et al. (2025). Although different in its implementation, it is still similar in spirit in the sense that the study also describes an avenue to factor out the main environmental effects in the context of penalized factorial regression.

The two designed target variables capture different components of the genotypic variance. While yHatGGE consists of both the GxE interaction effects and the genotypic main effect $\hat{g}_{i}$, yHatGE covers the GxE interaction effects only. Therefore, after inference $\hat{g}_{i}$ needs to be added to the predicted yHatGE for the final performance value. The difference between yHatGE + $\hat{g}_{i}$ and yHatGGE was small across CV schemes and evaluation metrics. Generally, we see the following limitations of the two designed GxE target variables. Both, yHatGE and yHatGGE are linear combinations of effect estimates from the model in Eq. (1) and not of the true effects. As illustrated in Fig. S3, the estimates of $\hat{g}_{i}$ and yHatGE from CV splits differ from those estimated using the full training dataset. The precision of the estimation varies depending on the training-set split. Fig. S3 shows a reduction in the accordance between LMM coefficient estimates from the full training set versus LMM coefficient estimates from the CV splits, decreasing from E/L to Y/LY. The lowest within-environment PCC values were observed for yHatGE in LY, with an average of about 0.9 between full training-set-based estimates and CV-split-specific training-set estimates. In some splits and environments PCC was as low as 0.3. Compared to yHatGE, $\hat{g}_{i}$ remained largely stable across CV schemes; however, a trend emerges when comparing E/L CV with Y/LY CV, where the number of splits exhibiting lower PCC for $\hat{g}_{i}$ increased. This illustrates that the target variables we provided to the ML and DNN models are not ground truth but are subject to estimation error. Furthermore, yHatGE and yHatGGE have different limitations. When we use yHatGGE as target variable, we indirectly re-estimate $\hat{g}_{i}$ via the ML or DNN models, which causes unnecessary additional prediction error and double shrinkage. On the other hand, predicted yHatGE has experienced double shrinkage, both as random term in LMM (1) fit and during ML/DNN model training. This is then combined with the random effect estimate $\hat{g}_{i}$, which was shrunk only once during LMM fit from Eq. (1). Thus, the combination of predicted yHatGE values with $\hat{g}_{i}$ is suboptimal because it indirectly increases the relative weight of $\hat{g}_{i}$. This is likely the effect we observe in Fig. S5, which shows that yHatGE + $\hat{g}_{i}$ predictions more strongly correlate with $\hat{g}_{i}$ than predictions of any other approach indicating that those predictions are dominated by $\hat{g}_{i}$. This is also supported by the improvement in MSED over $\hat{g}_{i}$ for yHatGE + $\hat{g}_{i}$ in Y and LY, which is almost zero for most environments (Figs. 5, 6). Generally, it would have been preferable to estimate all model terms in Eq. (1) used for yHatG(G)E as fixed effects (Holland and Piepho 2024). However, due to the excessive RAM requirements (> 128 GB) caused by the dataset size and the number of effects to be estimated, we were not able to fit such a model with the computational resources available to us.

Furthermore, our results showed that custom MSED-loss implementation substantially improved model performance compared to MSE loss, by up to 29%, 45% and 50% for LGBM, DNN and XGB, respectively (Figs. 3–6). As previously investigated in the field of human genetics, MSED-like measures can be successfully implemented into DNN model optimization (Drusinsky et al. 2024). In Y and LY, the improvement obtained by using MSED instead of the MSE loss was particularly strong for the DNN model (Figs. 5, 6), indicating that for XGB and LGBM the approach further refinement of the approach is required, e.g., by using the full Hessian, shown in Eq. (7), during optimization and thereby recovering within-environment genotype interactions. Nevertheless, results based on MSED loss showed a slight disadvantage in accuracy across all CV schemes and metrics compared to the two designed target variables (Figs. 3–6). The underlying reason for this behavior should be subject to further research.

Another idea, next to MSED loss, would be to use the within-environment PCC as loss function. Other researchers developed correlation-based loss functions, especially in conjunction with DNNs (Atmaja and Akagi 2021; Chen et al. 2022), which might be worthwhile to explore in GxE prediction as well. However, we consider the MSED as the more holistic optimization metric compared to PCC. In the following we would like to motivate our choice. In Table S5, we demonstrated that the MSED_*j*_ can be decomposed into three error components according to the decomposition presented by Gauch et al. (2003) for the MSE. Next to the correlation between true and predicted values within environments, the MSED_*j*_ does also capture rescaling effects, such as shrinkage. In contrast, PCC, by definition, is scale-invariant. For the decomposition, we first calculated pairwise differences between genotypes in the same environment, both from the set of predicted and the set of true target values and then calculated the MSE of such within-environment differences. Using the notation from Gauch et al. (2003) we defined $\Delta z_{j} = (z_{ij} -z_{i^{\prime}j}) _{{({i,i^{\prime}})}\in\,P{_j}}$ and $\Delta y_{j} = (y_{ij} -y_{i^{\prime}j}) _{{({i,i^{\prime}})}\in\,P{_j}}$, where *P*_*j*_ is the set of all genotype pairs in environment *j. *Then, we defined $z_{j} = \Delta z_{j} - \overline{\Delta z_{j}} ,\quad y_{j} = \Delta y_{j} - \overline{\Delta y_{j}}$, where $\overline{{{\Delta }z_{j}}}$ and $\overline{{{\Delta }y_{j}}}$ are the averages of the pairwise difference vectors for predicted and true values in environment *j*, respectively. Now the $SB = \left( {\overline{Z} - \overline{Y}} \right)^{2}$ in Gauch et al. (2003) becomes $SB_{j} = \left( {\overline{\Delta z_{j}} - \overline{\Delta y_{j}} } \right)^{2}$. Formulae for NU_*j*_ and LC_*j*_ remain the same as described in Gauch et al. (2003), since we have defined $z_{j}$ (in Gauch et al. (2003) termed $x_{n}$) and $y_{j}$ as vectors of deviations of predicted and true differences from the mean difference within environments. Their linear combination will yield the same MSED values as per Eq. (3) proposed by (Piepho 1998). The MSED captures rescaling in both SB and NU. The regression slope *b*, which is central to NU, can be expressed as $b_{j} = r_{j} \cdot \frac{{\sigma \left( {\Delta y_{j}} \right)}}{{\sigma \left( {\Delta z_{j}} \right)}}$, where $r_{j}$ is the PCC between the vectors of predicted and true pairwise genotype differences within environment *j* and $\frac{{\sigma \left( {\Delta y_{j}} \right)}}{{\sigma \left( {\Delta z_{j}} \right)}}$ is the ratio of the standard deviations of these vectors. This ratio effectively captures shrinkage of the predicted differences. We visualize its reciprocal in Fig. S6. The results indicate that each model imposed shrinkage on the predicted pairwise yield differences, albeit to differing degrees. Shrinkage was most pronounced for models trained on GDY with MSE loss. XGB and LGBM typically showed stronger shrinkage than the DNN, while the DNN and E-BLUP displayed similar levels. Additionally, shrinkage increased with the complexity of the CV scheme, from E to LY (Fig. S6).

The SCC, being based on ranks, is also not fully differentiable and therefore cannot be used as a loss function. Overall, we conclude that loss function engineering opens a promising avenue in GxE modeling and shows striking benefits compared to classical MSE/MAE objective functions. Furthermore, it has clear advantages over the two-stage target-variable-design approach in terms of speed and simplicity.

Lastly, we would like to briefly address the computational comparison among the evaluated methods. GxE modeling is classically dominated by LMM-based parametric models like factorial regression models, factor-analytic models or environmental-kinship-based LMM approaches (van Eeuwijk et al. 2016). However, as data volumes continue to grow rapidly, both in terms of observations and features, parametric models increasingly encounter convergence challenges and place significant demands on computational resources, including time and hardware. ML models, and to some extent DNN models, can offer an efficient and convenient alternative. They are typically fast, easy to implement and—depending on the model and its specific setup—might require less computational resources. As shown in Sect.  3.4, the proposed ML and DNN approaches showed drastically shorter computing times (Table 5). Compared to GBAs, DNNs require usually more computing time and memory. With ~ 23 k observations, the dataset used in this study represents a mid-size MET dataset. For larger MET dataset the computational burden of LMM-based models, like the E-BLUP model, can be prohibitive and may lead users to look for more resource-efficient models.

### Looking into the past to infer the future

Many relevant use cases ask for a prediction of genotype performances for future years. Growers, for instance, want to select the best-suited variety at their respective site for the next season (single target year). Similarly, a variety testing authority wants to give robust variety recommendations to growers valid for at least one year. Additionally, a portfolio manager is responsible for placing the most suitable variety for each specific market segment, typically making decisions that have implications over a multi-year period. And finally, also the breeder makes long- to mid-term decisions during population improvement and product development.

Our work showed that by using historical weather records, we could predict environment-specific genotype ranks by + 9.8% (Y) to 9.9% (LY) more accurately than a multi-year–location mean would have done (Figs. 5, 6). This means our suggested approaches improve the knowledge base for breeding and farming to make season-independent and more targeted decisions on, for instance product placement or variety selection in the future TPE. Similar to recent work by de los Campos et al. (2020), the developed framework can be used as the backbone of simulations of genotype differences in both the temporal and spatial dimension across the TPE. In line with this study, de los Campos et al. (2020) used historical weather data from years overlapping with their training set. Still, unlike our work, they excluded the spatial dimension. They produced genotype-by-location yield distributions based on previous years of weather data only for known trialing locations aiming to produce more robust location-specific genotype BLUEs than single- or two-year phenotypic trial data would deliver. In our study we aimed to predict year-specific genotype performances for unknown locations where genotype-by-location BLUEs are not available. Similar to what was described by Gillberg et al. (2019), as final prediction for the untested year we use the median value across single year predictions. In the future we also want to compare the prediction abilities based on historical weather data, with simulated future weather data predicted by general circulation models, e.g., CMIP6 (John et al. 2018), EURO-CORDEX (Jacob et al. 2014). We hypothesize that for short-term decision-making, e.g., a grower that selects a cultivar for the next season, historical-weather-based predictions are preferable, whereas the more long-term oriented a decision is, e.g., population development in breeding, the more likely the benefit of such climate forecast models becomes, but this should be subject to future investigation.

### General limitations

As stated in Materials and Methods Sect. 2.2.1, the trial-conducting institutions provided first-stage single-trial genotype BLUEs without their respective variance–covariance matrices, nor simple standard errors. Therefore, no weights were available to use in second-stage analysis. Running second-stage analysis unweighted, implicitly assumes all BLUEs are equally precise with constant error variance and independent, which is unrealistic. Consequently, high-error trial estimates might inflate the noise in second-stage estimates, which subsequently influence the constructed target variables yHatGE and yHatGGE. Möhring and Piepho (2009) compared weighted and unweighted two-stage analyses against a one-stage mixed-model benchmark and reported that a two-stage analysis without weighting would produce acceptable results with small trade-off compared to weighting with regards to the accuracy of the estimated genotype main effects. Generally, recovering the full variance–covariance matrices of the genotype BLUEs from individual trials estimated in first stage would have been the preferred approach (Piepho et al. 2012).

Another data-related limitation concerns the environmental data accuracy. In many cases, geographic coordinates were available only at a location level rather than for the exact experimental fields. Consequently, the derived environmental covariates may only approximate the conditions experienced by a trial and may not capture within-location heterogeneity, as we had explained in Sect. 2.1 of Materials and Methods. We expect this to be especially limiting for the predictive power of soil variables.

Additionally, key trial metadata, e.g., sowing dates and other management information were not consistently available. Ideally, the inclusion of this data, along with genotype-specific scores for key growth stages such as flowering time in hybrid rye (Riedesel et al. 2024), would have enabled the aggregation of environmental covariates into genotype-specific growth-stage windows, rather than relying on monthly aggregates. As demonstrated in Heslot et al. (2013), it is possible to use a crop growth model to determine trial-level growth stages even without phenology input. However, if an important part of GxE is driven by genotypic differences in phenology, e.g., early versus late flowering, shifting the timing of stress exposure (Shavrukov et al. 2017) and then applying the same window to all genotypes within a trial would blur that mechanism. We acknowledge that monthly aggregation, chosen due to data availability constraints, may be suboptimal.

Furthermore, no marker or pedigree data were available to relate individuals. In Section 4.4, we further describe how the lack of this information prevented us from evaluating cross-validation scenarios involving untested genotypes. Genomic marker data are generally not easily available for hybrid rye, which is a three- or even four-way hybrid with considerable levels of genetic variability of individual plants from the same variety. Alternatively, a classical pedigreed-based numerator relationship matrix A could be used in the future in hybrid rye GxE prediction. An elegant and simple method of incorporating pedigree information into ML and DNN models is to perform an eigendecomposition of the A matrix, followed by rank-k approximation $A \approx Z_{k} Z_{k}^{T}$, with $Z_{k} = U_{k} \Lambda_{k}^{1/2}$ where U and ${\Lambda }$ denote the eigenvectors and eigenvalues, respectively. The pedigree-based relatedness feature vector for genotype *i* is given by the *i*-th row of $Z_{k}$, $z_{i} = Z_{k} \left[ {i,:} \right] \in R^{k}$. This has been applied recently in the context of DNNs for instance by Fan and Waldmann (2025).

We did not control for multicollinearity among the weather covariates, nor did we compute variable importance measures. We acknowledge that some covariates are expected to be correlated, for example different summary statistics like mean, min and max of the same underlying weather variable but also solar radiation and temperature or precipitation and air humidity among others. For the following reasons, we expect correlated covariates to have limited impact on the predictive performance of the test models. Tree-based gradient boosting models like XGB and LGBM are generally robust to multicollinearity in terms of prediction, because splits are chosen to maximize impurity reduction rather than estimating linear coefficients. When predictors are highly correlated, trees typically use one of them for a split since additional, correlated predictors often provide little extra gain. In kernel-based LMM approaches like E-BLUP, covariates contribute via an environmental kernel rather than via explicit coefficient estimation. Therefore, the multicollinearity-induced instability of fixed-effect coefficients that can arise in linear regression is not directly relevant for the kernel-based E-BLUP approach. Nevertheless, redundant blocks of environmental covariates can lead to an overweighting of these predictors in the environmental relatedness kernel relative to less redundant signals. DNNs, can be sensitive to redundant inputs because correlated features reduce identifiability of weights and can encourage co-adaptation of representations. To mitigate this, we applied L2 regularization in every dense layer, incorporated drop-out layers and trained an ensemble of ten independently fitted networks to stabilize predictions and reduce overfitting. Future work could address multicollinearity more explicitly, including reduction of redundant predictors through, e.g., constructing synthetic covariates via principal component analysis (PCA; Wood 1976) of the full covariate set or partial least squares (PLS; Vargas et al. 1999).

### Applications

One major area of interest in the field of GxE modeling is to obtain a comprehensive understanding of the behavior of varieties across the TPE. The majority of studies designed CV schemes similar to the E scheme used in this study (Jarquín et al. 2014; Millet et al. 2019; de los Campos et al. 2020; Kick et al. 2023; Mumford et al. 2023; Ray et al. 2023; Avagyan et al. 2025; He et al. 2025). However, E only reflects scenarios where the user is interested in obtaining predictions for already observed year x location combinations. For example, if a breeder loses a trial location in a given year and seeks to impute the missing environment, this scheme is applicable. However, for most other scenarios, E tends to produce overly optimistic validation outcomes. This can be improved by using the L scheme that now allows the user to make statements about the models’ ability to predict completely new locations if the year of interest was part of the training set. It can be used to look retrospectively and roll out available MET observations to a full-picture view on the TPE based on environmental data. Studies which used L-type CV schemes include Washburn et al. (2021), Westhues et al. (2021), Montesinos-Lopez et al. (2022) or Tadese et al. (2024). On the other hand, Y CV can be used to better understand how genotypes might perform at known locations in new years without any observations in the training set (Westhues et al. 2021; Rogers and Holland 2022; Fernandes et al. 2024; Washburn et al. 2024). This is of interest when predicting known trialing locations retrospectively for years that occurred before any record in the training set or when predicting into the future although this entails another level of complexity since weather data are mostly not yet available when the prediction is of interest and hence additional methods have to be applied to approximate the likely future environmental condition at a given site as for instance the use of historical weather data (Gillberg et al. 2019; de los Campos et al. 2020) or climate model data. Finally, the combination of the L and Y scheme, the LY CV scheme often reflects practice most realistically and is underrepresented in the literature (Gillberg et al. 2019).

In this study, we demonstrated two novel methodological enhancements that improved the precision of environment-specific hybrid rye genotype rankings by + 15%, + 13.7%, + 9.8% and + 9.9% (median SCC) compared to rankings based on the genotype BLUPs $\hat{g}_{i}$ for E, L, Y and LY CV, respectively (Table S6). These four CV schemes depict the most relevant use cases for simulation of advanced material and varieties within the TPE. For the dataset at hand, a breeder could for instance impute genotype performances in lost trials with an average accuracy as high as 0.67 (E PCC; Fig. 3). A portfolio manager or a breeder could simulate rye genotype rankings retrospectively for the past 12 years for any random coordinate within the test set area with similarly high accuracy as for known locations (L; 0.656 median PCC; Fig. 4). Moreover, when predicting pre-season for any future TPE-environment based on historical weather data a grower, breeder or portfolio manager can expect moderate predictive abilities of 0.506 (LY PCC; Fig. 6). Both L and LY scenarios are highly relevant, and accurate models would allow stakeholders to broadcast knowledge seamlessly from MET network to TPE. While for the spatial dimension (L) we already obtained very promising results, the temporal dimension reflected in CV schemes Y and LY was still challenging. Figure S4 shows that the environmental similarity is substantially lower for environment pairs from different years compared to those from the same year. Although the proposed approaches do not model the pairwise genetic correlation between environments explicitly, they implicitly build upon environmental relatedness through environmental covariates, which induce correlated genotype responses across similar environments. This may explain to some extent why the prediction into untested years (Y and LY) generally yield much lower predictive accuracies compared to CV schemes like E and L. Similarly, Bustos-Korts et al. (2021) observed that environmental data from future years may be uncovered by the conditions captured in the training set inherently leading to lower predictive abilities (Malosetti et al. 2016; Rogers and Holland 2022). Generally, this highlights the need for future research to focus on tools that address the additional uncertainty associated with predicting future weather conditions.

We also observed that predictive abilities varied substantially between environments for any of the tested approaches with environment-wise PCCs ranging from −0.08 to 0.94 for E and L (Figs. 3, 4) and −0.32 to 0.94 for Y and LY (Figs. 5, 6) even for the best performing DNN model. This is a common concern in GxE prediction for untested environments (Heslot et al. 2013; Costa-Neto et al. 2022; Jung et al. 2025). There is no doubt that this still limits the robust application of GxE prediction in practice. Besides the endeavor of the research field to improve modeling accuracy, we also see great potential in the development of methods that seek to quantify the uncertainty associated with predictions and thereby allow the user to access the risk. Mixed-model theory offers well-defined concepts of standard errors for estimated means and pairwise differences of fixed effects, as well as standard errors of prediction for random effects and their contrasts. Bayesian frameworks, in turn, quantify uncertainty via posterior distributions rather than point estimates. By comparison, uncertainty quantification in machine-learning and deep neural network models is still less established. In future work we would like to address this problem and test methods like the Jackknife + suggested by Barber et al. (2021) and distribution-free rank-one-out conformal inference suggested by Lei et al. (2018).

Next to E, L, Y and LY other high-impact predictions scenarios, especially for breeding, would include the prediction of early-stage breeding material without phenotypic records in untested environments. All these scenarios obviously require information on the genetic make-up, which was not available in this study. Regardless of the CV scheme, a general advantage of information on genetic relatedness is the increase in data connectivity by allowing to borrow information between environments with diverse sets of genotypes.

As mentioned earlier, hybrid rye is known for its relatively stable behavior across a wide range of environments (Wilde and Miedaner 2021). In the dataset at hand, we observed that approximately 50% and 40% of genotype-related variance was explained by GxE interaction in training set and test set, respectively (Fig. 2). This is comparable to what has been reported for corn by Kick and Washburn (2023) in the USA and by Bocianowski et al. (2024) in Poland although crops, like wheat and corn, frequently were reported to show higher levels of GxE variance than rye (Utz and Laidig 1989). Laidig et al. (2017) reported five times higher levels of GxE than genotypic variance from a 26-years German OVT testing series on rye, but the comparison to our dataset is difficult due a substantial proportion of population varieties in this dataset as opposed to ours. Sub-structuring into regional programs like usually observed for wheat is not common in hybrid rye. However, climate change is increasing spatial heterogeneity of crop productivity inside the TPE (Challinor et al. 2014; Kersebaum and Nendel 2014) and is also presumed to significantly affect hybrid rye production (Ghafoor et al. 2024). Similarly, studies like Riedesel et al. (2024) advocate for a more site-specific breeding approach in rye. Overall, one of the major objectives of any commercial breeding program is to maximize seed sales. This can be achieved via 1) supplying a large TPE and 2) securing a high market share within that TPE. Traditionally breeding had answered this with selection for broad adaptation. Now, advances in the field of GxE modeling and abundancy of large-scale environmental covariates create the opportunity for plant breeding to apply narrow-adaptation selection in practice (Casadebaig et al. 2022; Bakare et al. 2024). One option is to partition the TPE into clusters, also called mega-environments that minimize within-cluster GxE (Chenu et al. 2013; Bustos-Korts 2017; Fradgley et al. 2025). Ideally, these should be stable across years, which has been reported to be a major challenge especially, when static (location-associated) GxE is small compared to non-static (year-dependent) GxE (Yan 2019; Bustos-Korts et al. 2022; Fradgley et al. 2025). Environment-specific predictions, like those developed in this study, could then be aggregated cluster-wise. Furthermore, for many growers, the primary concern is year-to-year risk at their respective location or region, rather than performance stability across the entire TPE. Here, environment-specific predictions can be used to produce within-cluster stability metrics to quantify the across-year variability of genotypes in a cluster and report this to the grower for better risk assessment.

## Conclusion

This study presents two new tools—target-variable engineering and MSED-loss-function engineering—that were shown to enhance the precision of environment-specific genotype ranking predictions in hybrid rye. Through extensive cross-validation, we showed that these approaches consistently outperform traditional ML/DNN methods that rely on MSE-based optimization and even parametric environmental-kinship-based LMMs in many cases, achieving prediction gains of up to + 15% over baseline genotype means $\hat{g}_{i}$.

Our work highlights that by explicitly focusing on GxE effects during model training, ML and DNN models can effectively bridge the gap between classical statistical models and scalable, high-throughput predictive frameworks needed for modern plant breeding. Furthermore, we demonstrated that leveraging historical weather records improves more robust prediction of genotype performances for untested years, opening new opportunities for decision-making under future uncertainty in breeding and farming. Overall, the methodologies developed herein not only advance state-of-the-art GxE prediction in hybrid rye but also lay the foundation for broader applications across crops.

## Supplementary Information

Below is the link to the electronic supplementary material.

Supplementary file1 (PDF 941 KB)

## Declarations

### Data Availability

Data were provided by KWS SAAT SE & Co. KGaA, BSA, COBORU and AGES for exclusive use in this study and is generally not publicly available. Reasonable requests may be addressed to the respective organization. Data from Danish OVTs and PRTs, from Czech OVTs and from German PRTs are publicly available as described in Table S1.
