# Supplementary material for: Tailoring AI and ML models for genotype-by-environment prediction leveraging environmental covariates: A European rye example
Source: Theor Appl Genet. 2026 Jul 15;139(8):206. doi: 10.1007/s00122-026-05280-z (PMC13372854; doi:10.1007/s00122-026-05280-z)
Supplement: Supplementary file 1 — Supplementary file1 (PDF 941 KB) [file 122_2026_5280_MOESM1_ESM.pdf]

Supplementary material

Title

Tailoring AI and ML Models for Genotype-by-Environment Prediction Leveraging Environmental Covariates: A European Rye Example

Journal name

Theoretical and Applied Genetics

Authors

Wera Eckhoff[1,2], Florence Parat[2], Gennady Bracho-Mujica[2], Clemens Flamm[3], Daniela Bustos-Korts[4], Hans-Peter Piepho[1]

Affiliations

[1] Biostatistics Unit, Institute of Crop Science, University of Hohenheim, Stuttgart, Germany

[2] KWS SAAT SE & Co. KGaA, Einbeck, Germany

[3] Österreichische Agentur für Gesundheit und Ernährungssicherheit GmbH, Wien, Österreich

[4] Faculty of Agricultural and Food Sciences, Universidad Austral de Chile, Valdivia, Chile

E-Mail

Wera Eckhoff, Orcid-ID: 0000-0002-5639-9757, w.eckhoff@uni-hohenheim.de (Corresponding author)

Hans-Peter Piepho: hans-peter.piepho@uni-hohenheim.de

Daniela Bustos-Korts: daniela.bustos@uach.cl

Table S1 Overview of phenotypic data authors and sources used as training set, where OVT stands for official variety trials, PRT stands for post-release trials and VP stands for KWS precommercial trials. Country abbreviations Austria (AT), Czech Republic (CZ), Germany (DE), Denmark (DK) and Poland (PL).

**Country**	**Trial**	**Author**	**Publicly available**	**Database address**
AT	OVT	Austrian Agency for Health and Food Safety (AGES)	no	-
CZ	OVT	Central Institute for Supervising and Testing in Agriculture (ÚKZÚZ)	yes	https://ukzuz.gov.cz/public/portal/ukzuz/en/
DE	OVT	German Federal Office of Plant Varieties (BSA)	no	https://www.bundessortenamt.de/bsa/antragsteller/berichte-der-wertpruefung
DK	OVT	TystofteFonden	yes	https://sortinfo.dk/#/overview/88011230/latestYear/VP
PL	OVT	Research Center for Cultivar Testing (COBORU)	no	https://www.coboru.gov.pl/pdo/pdoPublikacjeRegionalne
AT	PRT	AGES/ Land-NÖ[2]	yes	https://lako.at/versuche/
DE	PRT	German Federal Agricultural Chambers[1]	yes	separate ‘Landessortenversuche’ reports from webpage of respective Chamber
DK	PRT	TystofteFonden/ SEGES Innovation	yes	https://sortinfo.dk/#/overview/88011230/latestYear/LF
DE/PL	VP	KWS SAAT SE & Co. KGaA	no	-

[1] *Landwirtschaftskammer Schleswig-Holstein (LKSH); Landesforschungsanstalt für Landwirtschaft und Fischerei Mecklenburg-Vorpommern (LFA); Landesamt für Ländliche Entwicklung, Landwirtschaft und Flurneuordnung Brandenburg (LELF); Landesanstalt für Landwirtschaft und Gartenbau Sachsen-Anhalt (LLG); Thüringer Landesamt für Landwirtschaft und Ländlichen Raum (TLLLR); Sächsisches Landesamt für Umwelt, Landwirtschaft und Geologie (LFULG), Dienstleistungszentrum Ländlicher Raum Rheinland-Pfalz (DLR); Landwirtschaftskammer Nordrhein-Westfalen (LWK NRW); Bayrische Landesanstalt für Landwirtschaft (LfL); Landesbetrieb Landwirtschaft Hessen (LLH); Landwirtschaftskammer Niedersachsen (LWK NDS); Landwirtschaftliches Technologiezentrum Augustenberg (LTZ);* [2]*In Lower-Austria (Land-NÖ) PRT trials are conducted by agricultural schools.*

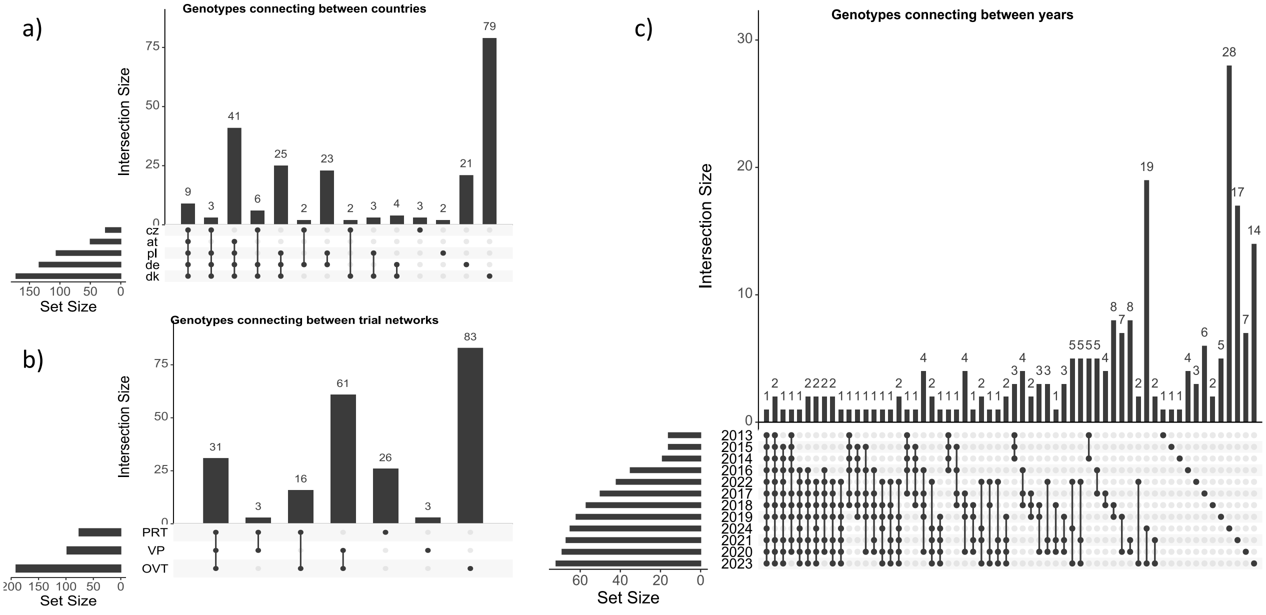

Fig. S1 Connectivity between genotypes and a) Countries, b) trial networks (PRT = post-release trials, VP = KWS precommercial trials, OVT = official variety trials) and c) years. Country abbreviations Austria (AT), Czech Republic (CZ), Germany (DE), Denmark (DK) and Poland (PL).

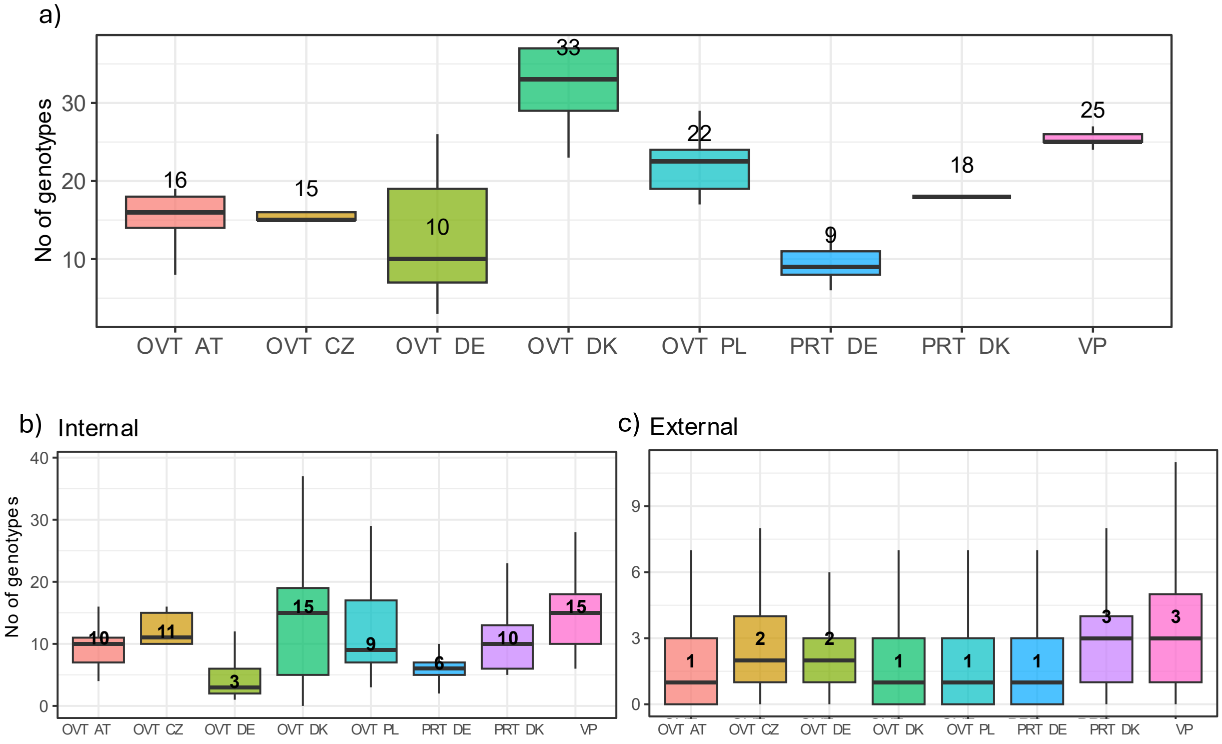

Fig. S2 Overview on a) number of unique genotypes per trial in each trial network and number of intersecting genotypes between b) trials of the same trial network and c) trials from distinct trial networks. Country abbreviations Austria (AT), Czech Republic (CZ), Germany (DE), Denmark (DK) and Poland (PL).

Table S2 Overview on hyperparameter spaces used during optuna hyperparameter tuning for machine leaning and deep learning models used in this study.

Hyperparameter	Distribution	Space
**XGBoost**		
lambda	loguniform	1e-8, 10.0
alpha	loguniform	1e-8, 10.0
max_depth	int	3, 10
eta	loguniform	0.01, 0.3
subsample	uniform	0.5, 1.0
colsample_bytree	uniform	0.5, 1.0
num_boost_round	int	50, 5000
**LightGBM**		
lambda_l1	loguniform	1e-8, 10.0
lambda_l2	loguniform	1e-8, 10.0
num_leaves	int	2, 256
feature_fraction	uniform	0.6, 1.0
bagging_fraction	uniform	0.4, 1.0
bagging_freq	int	1, 7
min_child_samples	int	5, 100
max_depth	int	4, 16
learning_rate	loguniform	0.001, 0.1
min_data_in_leaf	int	20, 300
num_boost_round	int	20, 2000
**Deep Neural Network**		
n_neurons_layer_1	int	1000, 4000
n_neurons_layer_2	int	200, 3000
n_neurons_layer_3	int	100, 2000
n_neurons_layer_4	int	50, 500
n_neurons_layer_5	int	20, 300
dropout_rate_1	uniform	0.0001, 0.6

dropout_rate_2	uniform	0.0001, 0.6
learning_rate	loguniform	0.00001, 0.02
l2_reg1	loguniform	1e-6, 1e-1
l2_reg2	loguniform	1e-6, 1e-1
batch_size	categorical	2048, 4096, 8192, 16384, 32768
epochs	categorical	100, 150, 200

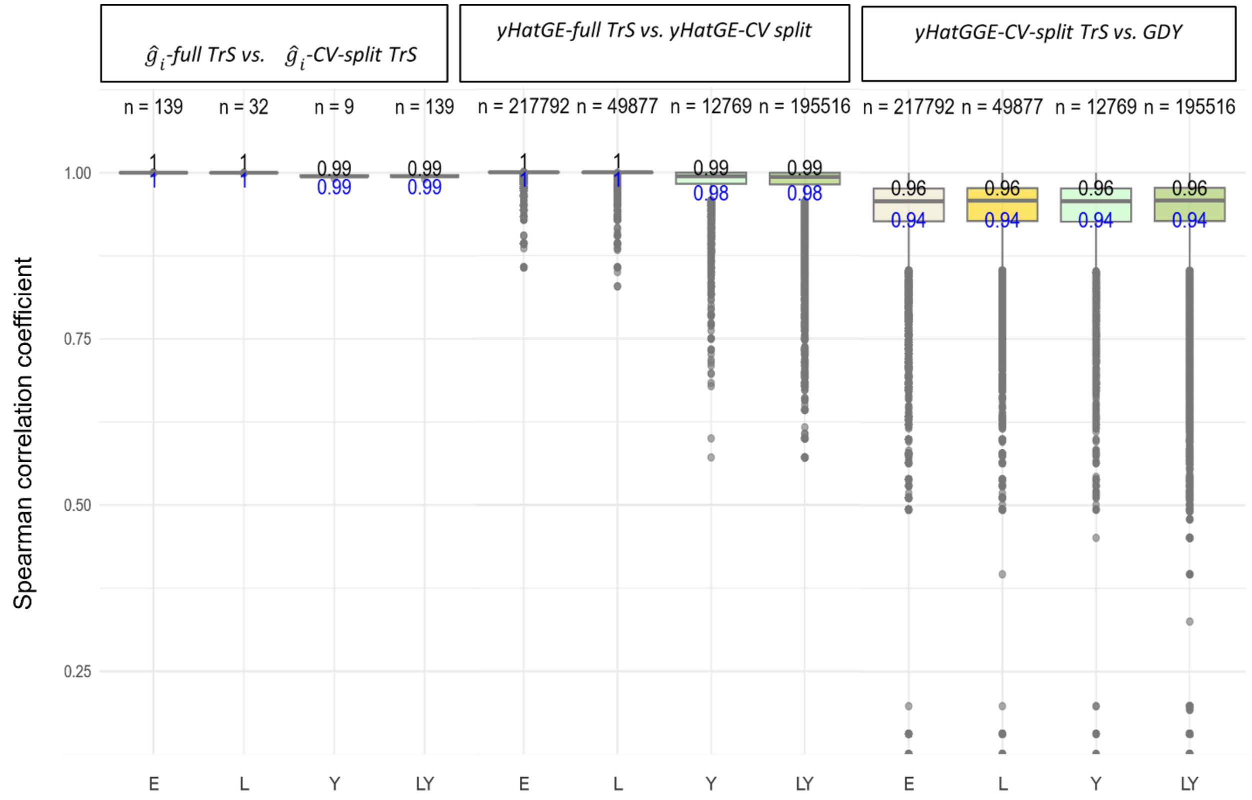

Fig. S3 Boxplot of within-environment Spearman correlation coefficients (SCC) between 1) $\hat{\boldsymbol{g}}_{\boldsymbol{i}}$estimated from the full training set (TrS) and $\hat{\boldsymbol{g}}_{\boldsymbol{i}}$ estimated from TrS of individual cross-validation (CV) splits, 2) between yHatGE estimated from the full TrS and yHatGE estimated from TrS of individual CV splits and 3) between yHatGGE estimated from TrS of individual CV splits and grain dry matter yield (GDY). N represents the number of SCC values within each boxplot. The designed target variables, yHatGE and yHatGGE, are defined as linear combinations of estimated coefficients derived from the variance components decomposition of GDY. The x-axis represents the cross-validation schemes, which include leave-one-envCV-out (E), leave-one-location-out (L), leave-one-year-out (Y), and leave-one-year-and-one-location-out (LY). EnvCV represents the unique combination of year and location. Values in blue and black text represent the mean and median values, respectively.

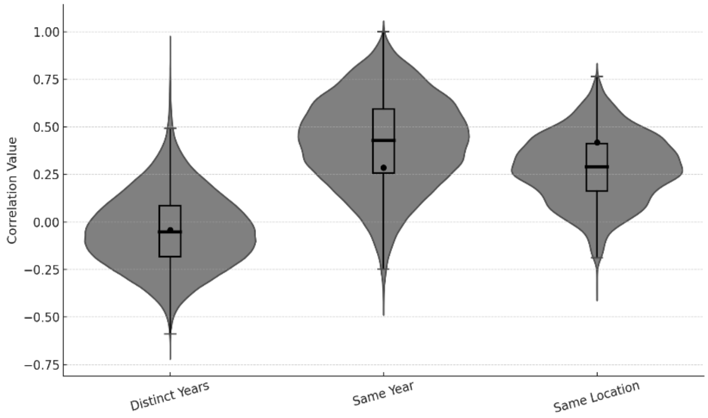

Fig. S4 Distributions of pair-wise environmental correlations (similarities). Kinship matrix E (Equation 4) was converted to correlation matrix for this visualization.

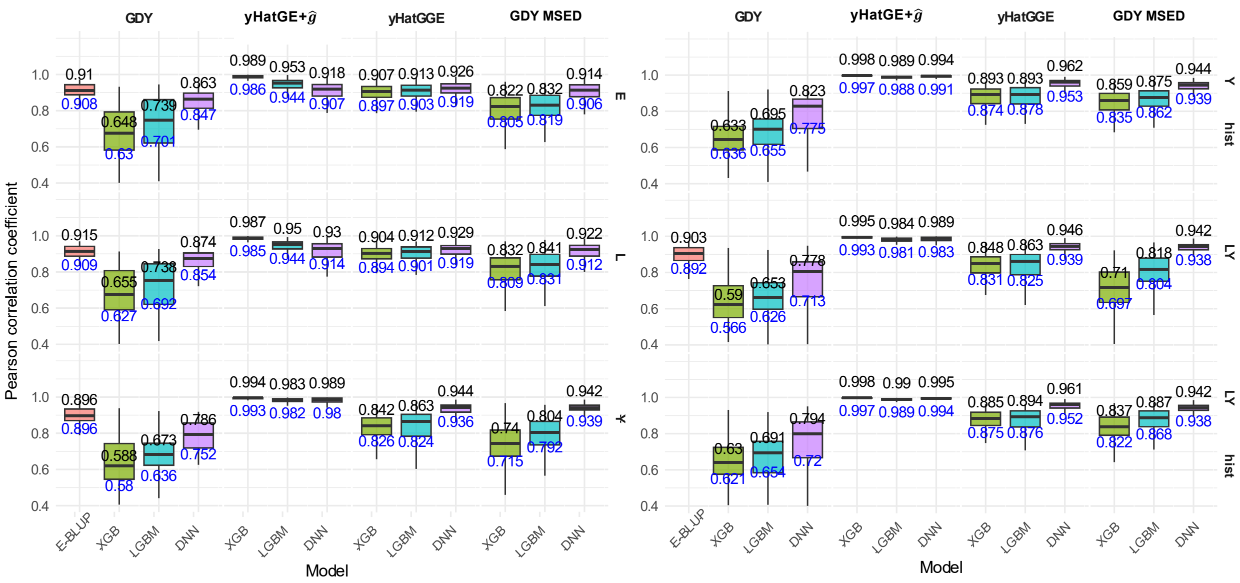

Fig. S5 Within-environment Pearson correlation values between $\hat{g}_i$ of the respective cross-validation (CV) split and predicted values from different modeling approaches defined as the combination between target variable plus loss function as described by the labels in the top row and bottom row. The type of CV scheme is indicated by the labels on the right side of the plots where "hist" indicates that the prediction was based on historical weather records. The CV schemes include leave-one-envCV-out (E), leave-one-location-out (L), leave-one-year-out (Y), and leave-one-year-and-one-location-out (LY). EnvCV represents the unique combination of year and location. The models included an environmental-kinship-based BLUP model adapted from Jarquín et al. (2014) (E-BLUP), two extreme gradient boosting algorithms XGBoost (XGB) and LightGBM (LGBM), and a deep neural network (DNN). The target variables included grain dry matter yield (GDY) and two constructed target variables, namely yHatGE and yHatGGE, which are defined as linear combinations of estimated coefficients derived from the variance components decomposition of GDY. After prediction, $\hat{g}_i$ was added to yHatGE. Within-environment mean squared error of differences (MSED) was used as loss-function, and the corresponding results are labeled accordingly. Values in blue and black text represent the mean and median values, respectively.

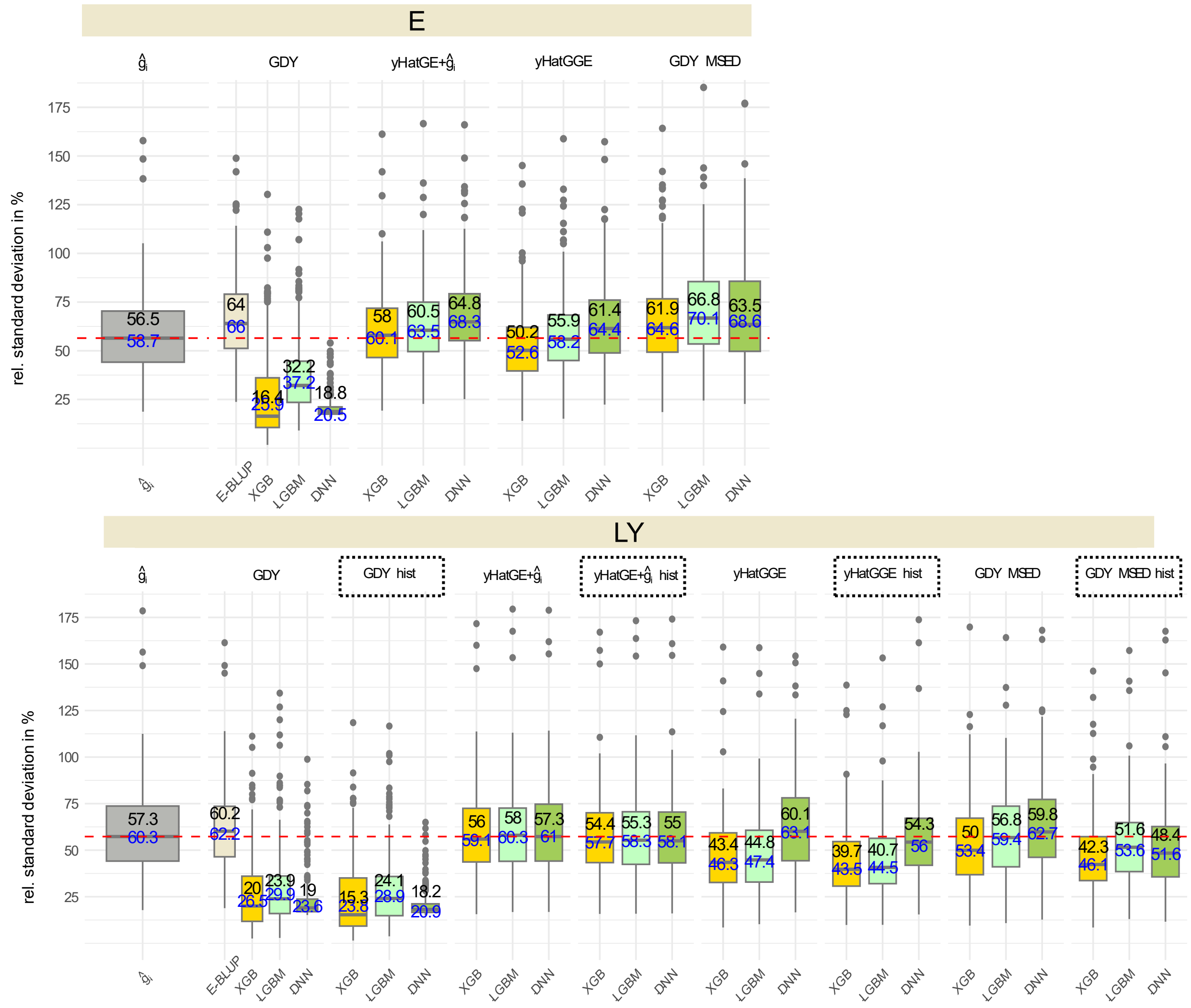

Fig. S6: Relative standard deviation (%) of predicted pairwise genotype differences compared to true pairwise genotype differences within environments for two cross-validation schemes, which include leave-one-envCV-out (E) and leave-one-year-and-one-location-out (LY). Top row labels indicate the target-loss combination used. EnvCV represents the unique combination of year and location. Labels in boxes refer to predictions based on historical weather records. Bottom row labels and coloring indicate which model was used, which include an environmental-kinship-based BLUP model adapted from Jarquín et al. (2014) (E-BLUP), two extreme gradient boosting algorithms XGBoost (XGB) and LightGBM (LGBM), and a deep neural network (DNN). The target variables included grain dry matter yield (GDY) and two constructed target variables, namely yHatGE and yHatGGE, which are defined as linear combinations of estimated coefficients derived from the variance components decomposition of GDY. After prediction, $\hat{\boldsymbol{g}}_{\boldsymbol{i}}$ was added to yHatGE. Within-environment mean squared error of differences (MSED) was used as loss-function, and the corresponding results are labeled accordingly. If the target label is followed by “hist” this indicates that the prediction was made based on historical weather records. Values in blue and black text represent the mean and median values, respectively.

Table S3: List of dynamic environment variables. Each variable was retrieved on daily basis and aggregated to monthly means.

Number	Variable	Unit	Description
1	air_temp_avg	Celsius	Average daily air temperature at two meters above ground level.
2	air_temp_max	Celsius	Maximum daily air temperature at two meters above ground level.
3	air_temp_min	Celsius	Minimum daily air temperature at two meters above ground level.
4	dew_point_avg	Celsius	Average dew point temperature data.
5	dew_point_max	Celsius	Maximum dew point temperature data.
6	dew_point_min	Celsius	Minimum dew point temperature data.
7	precip_acc_period_adjusted	millimeter	Amount of liquid-equivalent precipitation, fundamentally derived from the raw precipitation product, but then adjusted to more closely match available ground truth observations.
8	relative_humidity_avg	-	Average relative humidity data.
9	relative_humidity_max	-	Maximum relative humidity data.
10	relative_humidity_min	-	Minimum relative humidity data.
11	long_wave_radiation_avg	Watt per square meter	Average downwelling longwave radiation flux data.
12	long_wave_radiation_max	Watt per square meter	Maximum downwelling longwave radiation flux data.
13	long_wave_radiation_min	Watt per square meter	Minimum downwelling longwave radiation flux data.
14	short_wave_radiation_avg	Watt per square meter	Average downwelling shortwave radiation flux data.
15	short_wave_radiation_max	Watt per square meter	Maximum downwelling shortwave radiation flux data.
16	sunshine_duration	hours	Number of hours of sunshine based on the amount of cloud cover and downwelling shortwave radiation over the course of the day.

17	abs_scaled_paw_soil_moisture_0to10cm		Moisture content of the layer between the surface and the 10-cm depth expressed as a scaled value between field capacity (1.0) and wilting point (0.0) for the soil type at the chosen location.
18	abs_scaled_paw_soil_moisture_0to200cm		See 17 for 0 to 200 cm depth
19	normalized_soil_moisture_ 0to10cm		Provides an estimate of how the soil moisture level compares with the location's climatological average value in the layer between the surface and the 10-cm depth. A value of 0.5 (-0.5) indicates that the moisture level at the indicated time and location is 0.5 standard deviations above (below) the normal location's climatological average soil moisture level for the same layer, location, and time.
20	normalized_soil_moisture_ 0to200cm		See 19 for 0 to 200 cm depth
21	soil_moisture_0to10cm	millimeter	Total moisture content of the layer between the surface and the 10-cm depth.
22	soil_moisture_0to200cm	millimeter	See 21 for 0 to 200 cm depth
23	soil_temp_0to10cm	Celsius	Average temperature of the layer between the surface and the 10-cm depth
24	soil_temp_max_0to10cm	Celsius	Maximum temperature of the layer between the surface and the 10-cm depth
25	soil_temp_min_0to10cm	Celsius	Minimum temperature of the layer between the surface and the 10-cm depth

*Extended information at https://docs.clearag.com/documentation/Weather_Data/Historical_and_Climatological_Weather/latest#_response_object_json_5

Table S4: List of static soil variables. Each variable was available in 5 consecutive depth intervals (0–5 cm, 5–15 cm, 15–30 cm, 30–60 cm, 60–100 cm).

Number	Variable	Unit	Description
1	cec	cmol(c)/kg	Cation exchange capacity
2	cfvo	cm3/100cm3 (vol%)	Volumetric fraction of coarse fragments (> 2 mm)
3	clay	g/100g (%)	Proportion of clay particles (< 0.002 mm) in the fine earth fraction
4	sand	g/100g (%)	Proportion of sand particles (> 0.05/0.063 mm) in the fine earth fraction
5	silt	g/100g (%)	Proportion of silt particles (≥ 0.002 mm and ≤ 0.05/0.063 mm) in the fine earth fraction
6	phh2o	pH	Soil pH
7	soc	g/kg	Soil organic carbon content in the fine earth fraction
8	wv0010		Volumetric Water Content at 10kPa
9	wv0033		Volumetric Water Content at 33kPa
10	wv1500		Volumetric Water Content at 1500kPa

*Extended information at [SoilGrids — global gridded soil information | ISRIC]

Table S5: Illustrative example sets of true and predicted values to demonstrate the decomposition of the mean squared error of differences (MSED) into three error components (SB, NU and LC) according to Gauch et al. (2003). Columns y and x show the true and predicted target values.

	**Per-se values**			**Differences**		
**Set**	**y**	**x**		**Pair**	**Δy**	**Δx**
Absolute shift	60	80		60–90	-30	-30
Absolute shift	90	110		60–110	-50	-50
Absolute shift	110	130		90–110	-20	-20
Shrinkage	60	75		60–90	-30	-10
Shrinkage	90	85		60–110	-50	-20
Shrinkage	110	95		90–110	-20	-10
Scatter	60	80		60–90	-30	-2

Scatter	90	82		60–110	-50	-20
Scatter	110	100		90–110	-20	-18
Rank change	60	90		60–90	-30	30
Rank change	90	60		60–110	-50	-20
Rank change	110	110		90–110	-20	-50
Rescale of differences	60	60		60–90	-30	-3
Rescale of differences	90	63		60–110	-50	-5
Rescale of differences	110	65		90–110	-20	-2

**Metrics**						
**Set**	**PCC**[1]	**MSED**[2]	**SB**	**NU**	**LC**	**MSED_Gauch**[3]
Absolute shift	1	0	0	0	0	0
Rescale of differences	1	1026	900	126	0	1026
Shrinkage	0.99	466.67	400	50	16.67	466.67
Scatter	0.85	562.67	400	19.97	142.69	562.67
Rank change	0.29	1800	400	1250	150	1800

[1] PCC = Pearson correlation coefficient; [2] MSED calculated according to Piepho (1998); [3] MSED calculated as the linear combination of the three error components SB, NU and LC according to Gauch et al. (2003).

Table S6: Cross-validation results measured as within-environment Spearman rank correlation of tested approaches. The CV schemes include leave-one-envCV-out (E), leave-one-location-out (L), leave-one-year-out (Y), and leave-one-year-and-one-location-out (LY). EnvCV represents the unique combination of year and location. $\hat{\mathbf{g}}_\mathbf{i}$, the genotype main effect estimated as best linear unbiased prediction (BLUP) serves a lower benchmark. Four different models in combination with three different target variables and using the mean squared error of differences (MSED) as the loss function were compared. The models included an environmental-kinship-based BLUP model adapted from Jarquín et al. (2014) (E-BLUP), two extreme gradient boosting algorithms XGBoost (XGB) and LightGBM (LGBM), and a deep neural network (DNN). The target variables included grain dry matter yield (GDY) and two constructed target variables, yHatGE and yHatGGE, which are defined as linear combinations of estimated coefficients related to $\hat{\mathbf{g}}_\mathbf{i}$, and its interaction with the environment derived from the variance components decomposition of GDY. After prediction, $\hat{\boldsymbol{g}}_{\boldsymbol{i}}$ was added to yHatGE.

			Within-environment Spearman rank correlation												
Target variable approach	Model	CV	Mean	Median	%-delta to mean $\hat{g}_i$	%-delta to median $\hat{g}_i$	%-delta to mean E-BLUP	%-delta to median E-BLUP	CV	Mean	Median	%-delta to mean $\hat{g}_i$	%-delta to median $\hat{g}_i$	%-delta to mean E-BLUP	%-delta to median E-BLUP
GDY	$\hat{g}_i$	E	0.507	0.547	0.00	0.00	-11.05	-10.47	L	0.507	0.545	0.00	0.00	-11.21	-12.38
GDY	E-BLUP	E	0.57	0.611	12.43	11.70	0.00	0.00	L	0.571	0.622	12.62	14.13	0.00	0.00
GDY	XGB	E	0.361	0.369	-28.80	-32.54	-36.67	-39.61	L	0.37	0.371	-27.02	-31.93	-35.20	-40.35
GDY	LGBM	E	0.421	0.443	-16.96	-19.01	-26.14	-27.50	L	0.427	0.453	-15.78	-16.88	-25.22	-27.17
GDY	DNN	E	0.448	0.487	-11.64	-10.97	-21.40	-20.29	L	0.47	0.487	-7.30	-10.64	-17.69	-21.70
yHatGE+$\hat{g}_i$	XGB	E	0.546	0.581	7.69	6.22	-4.21	-4.91	L	0.546	0.589	7.69	8.07	-4.38	-5.31
yHatGE+$\hat{g}_i$	LGBM	E	0.571	0.618	12.62	12.98	0.18	1.15	L	0.565	0.614	11.44	12.66	-1.05	-1.29
yHatGE+$\hat{g}_i$	DNN	E	0.59	0.629	16.37	14.99	3.51	2.95	L	0.575	0.622	13.41	14.13	0.70	0.00
yHatGGE	XGB	E	0.532	0.58	4.93	6.03	-6.67	-5.07	L	0.533	0.569	5.13	4.40	-6.65	-8.52
yHatGGE	LGBM	E	0.547	0.594	7.89	8.59	-4.04	-2.78	L	0.548	0.597	8.09	9.54	-4.03	-4.02
yHatGGE	DNN	E	0.578	0.617	14.00	12.80	1.40	0.98	L	0.58	0.623	14.40	14.31	1.58	0.16
GDY MSED	XGB	E	0.514	0.555	1.38	1.46	-9.82	-9.17	L	0.515	0.579	1.58	6.24	-9.81	-6.91
GDY MSED	LGBM	E	0.557	0.603	9.86	10.24	-2.28	-1.31	L	0.552	0.596	8.88	9.36	-3.33	-4.18
GDY MSED	DNN	E	0.576	0.623	13.61	13.89	1.05	1.96	L	0.567	0.609	11.83	11.74	-0.70	-2.09
GDY	$\hat{g}_i$	Y	0.408	0.427	0.00	0.00	-5.56	-8.37	LY	0.406	0.424	0.00	0.00	-4.92	-6.40
GDY	E-BLUP	Y	0.432	0.466	5.88	9.13	0.00	0.00	LY	0.427	0.453	5.17	6.84	0.00	0.00
GDY	XGB	Y	0.27	0.294	-33.82	-31.15	-37.50	-36.91	LY	0.28	0.299	-31.03	-29.48	-34.43	-34.00
GDY	LGBM	Y	0.32	0.352	-21.57	-17.56	-25.93	-24.46	LY	0.314	0.339	-22.66	-20.05	-26.46	-25.17
GDY	DNN	Y	0.288	0.295	-29.41	-30.91	-33.33	-36.70	LY	0.291	0.309	-28.33	-27.12	-31.85	-31.79

yHatGE+$\hat{g}_i$	XGB	Y	0.414	0.442	1.47	3.51	-4.17	-5.15	LY	0.411	0.449	1.23	5.90	-3.75	-0.88
yHatGE+$\hat{g}_i$	LGBM	Y	0.418	0.446	2.45	4.45	-3.24	-4.29	LY	0.411	0.452	1.23	6.60	-3.75	-0.22
yHatGE+$\hat{g}_i$	DNN	Y	0.419	0.445	2.70	4.22	-3.01	-4.51	LY	0.411	0.44	1.23	3.77	-3.75	-2.87
yHatGGE	XGB	Y	0.383	0.385	-6.13	-9.84	-11.34	-17.38	LY	0.38	0.414	-6.40	-2.36	-11.01	-8.61
yHatGGE	LGBM	Y	0.372	0.387	-8.82	-9.37	-13.89	-16.95	LY	0.375	0.41	-7.64	-3.30	-12.18	-9.49
yHatGGE	DNN	Y	0.428	0.467	4.90	9.37	-0.93	0.21	LY	0.427	0.458	5.17	8.02	0.00	1.10
GDY MSED	XGB	Y	0.336	0.344	-17.65	-19.44	-22.22	-26.18	LY	0.329	0.371	-18.97	-12.50	-22.95	-18.10
GDY MSED	LGBM	Y	0.361	0.372	-11.52	-12.88	-16.44	-20.17	LY	0.343	0.366	-15.52	-13.68	-19.67	-19.21
GDY MSED	DNN	Y	0.421	0.461	3.19	7.96	-2.55	-1.07	LY	0.417	0.452	2.71	6.60	-2.34	-0.22
GDY	XGB	Y_hist	0.317	0.339	-22.30	-20.61	-26.62	-27.25	LY_hist	0.299	0.334	-26.35	-21.23	-29.98	-26.27
GDY	LGBM	Y_hist	0.334	0.347	-18.14	-18.74	-22.69	-25.54	LY_hist	0.33	0.348	-18.72	-17.92	-22.72	-23.18
GDY	DNN	Y_hist	0.3	0.316	-26.47	-26.00	-30.56	-32.19	LY_hist	0.3	0.322	-26.11	-24.06	-29.74	-28.92
yHatGE+$\hat{g}_i$	XGB	Y_hist	0.421	0.45	3.19	5.39	-2.55	-3.43	LY_hist	0.418	0.452	2.96	6.60	-2.11	-0.22
yHatGE+$\hat{g}_i$	LGBM	Y_hist	0.431	0.466	5.64	9.13	-0.23	0.00	LY_hist	0.423	0.463	4.19	9.20	-0.94	2.21
yHatGE+$\hat{g}_i$	DNN	Y_hist	0.429	0.456	5.15	6.79	-0.69	-2.15	LY_hist	0.422	0.458	3.94	8.02	-1.17	1.10
yHatGGE	XGB	Y_hist	0.404	0.427	-0.98	0.00	-6.48	-8.37	LY_hist	0.402	0.435	-0.99	2.59	-5.85	-3.97
yHatGGE	LGBM	Y_hist	0.395	0.425	-3.19	-0.47	-8.56	-8.80	LY_hist	0.387	0.42	-4.68	-0.94	-9.37	-7.28
yHatGGE	DNN	Y_hist	0.431	0.469	5.64	9.84	-0.23	0.64	LY_hist	0.429	0.466	5.67	9.91	0.47	2.87
GDY MSED	XGB	Y_hist	0.4	0.408	-1.96	-4.45	-7.41	-12.45	LY_hist	0.385	0.411	-5.17	-3.07	-9.84	-9.27
GDY MSED	LGBM	Y_hist	0.405	0.432	-0.74	1.17	-6.25	-7.30	LY_hist	0.384	0.404	-5.42	-4.72	-10.07	-10.82
GDY MSED	DNN	Y_hist	0.429	0.469	5.15	9.84	-0.69	0.64	LY_hist	0.423	0.45	4.19	6.13	-0.94	-0.66
